# Age and sex differences in efficacy of treatments for type 2 diabetes: A network meta-analysis

**DOI:** 10.1001/jama.2024.27402

**Published:** 2025-03-25

**Authors:** Peter Hanlon, Elaine Butterly, Lili Wei, Heather Wightman, Saleh Ali M Almazam, Khalid Alsallumi, Jamie Crowther, Ryan McChrystal, Heidi Rennison, Katherine Hughes, Jim Lewsey, Robert Lindsay, Stuart McGurnaghan, John Petrie, Laurie A Tomlinson, Sarah Wild, Amanda Adler, Naveed Sattar, David M Phillippo, Sofia Dias, Nicky J Welton, David A McAllister

**Affiliations:** 1 School of Health and Wellbeing, University of Glasgow, Glasgow, UK; 2 Institute of Genetics and Molecular Medicine, University of Edinburgh, Edinburgh, UK; 3 Department of Diabetes, Glasgow Royal Infirmary, NHS Greater Glasgow and Clyde, Glasgow, Glasgow, UK; 4 University of Glasgow BHF Glasgow Cardiovascular Research Centre, Glasgow, Glasgow, UK; 5 Epidemiology and Population Health, London School of Hygiene and Tropical Medicine, London, UK; 6 Usher Institute, University of Edinburgh, Edinburgh, UK; 7 Diabetes Trials Unit, University of Oxford, Oxford, UK; 8 Institute of Cardiovascular and Medical Sciences, University of Glasgow, Glasgow, UK; 9 Population Health Sciences, Bristol Medical School, University of Bristol, Bristol, UK; 10 Centre for Reviews and Dissemination, University of York, York, UK

## Abstract

**Importance:**

Sodium glucose cotransporter 2 inhibitors (SGLT2i), glucagon-like peptide-1 receptor analogues (GLP1ra) and dipeptidyl peptidase-4 inhibitors (DPP4i) improve hyperglycemia, and SGLT2i and GLP1ra reduce the risk of major adverse cardiovascular events (MACE) in patients with type 2 diabetes. It is not clear whether efficacy varies by age or sex.

**Objective:**

Assess whether age or sex are associated with differences in efficacy of SGL2i, GLP1ra and DPP4i.

**Data sources:**

Medline, Embase, trial registries.

**Study selection:**

Two reviewers screened for randomized controlled trials of SGLT2i, GLP1ra, or DPP4i compared with placebo/active comparator, in adults with type 2 diabetes.

**Data extraction and synthesis:**

We used individual participant data and aggregate-level data to estimate age-treatment and sex-treatment interactions in Bayesian multi-level network meta-regressions.

**Main Outcome and Measures:**

HbA1c and MACE

**Results:**

We identified 601 eligible trials [592 trials with 309,503 participants reporting HbA1c, mean age 59.0, SD (10.7) years, 43.1% female; 23 trials with 168,489 participants reporting MACE, mean age 64.0, SD (8.6) years, 44.0% female] and obtained individual participant data for 103 trials (103 reporting HbA1c and 6 reporting MACE). For SGLT2i, the magnitude of HbA1c reduction versus placebo was attenuated in older compared with younger participants (absolute reduction 0.24%; 95% credible interval (CrI) 0.10-0.38, 0.17%; 95% CrI 0.10-0.24 and 0.25%; 95% CrI 0.20-0.30 less HbA1c lowering per 30-year increment in age for monotherapy, dual therapy, and triple therapy, respectively). GLP1ra was associated with greater absolute HbA1c lowering with increasing age in monotherapy and dual-therapy (-0.18%; 95% CrI -0.31 to -0.05 and -0.24%; 95% CrI -0.40 to -0.07 HbA1c lowering per 30-yer increment respectively) but not triple therapy (0.04%; 95% CrI -0.02 to 0.11 per 30-year increment). DPP-4i was associated with slightly better absolute HbA1c lowering in dual-therapy for older people (-0.09%; 95% CrI -0.15 to -0.03 HbA1c lowering per 30-year increment), but the 95% CrIs included the null for mono and triple therapy (-0.08%; 95% CrI -0.18 to 0.01 and -0.01%; 95% CrI -0.06 to 0.05 respectively). The relative reduction in MACE with SGLT2i was greater in older compared with younger participants (HR 0.76; 95% CrI 0.62-0.93 per 30-year increment in age), whereas the opposite was found with GLP1ra (HR 1.47; 95% CrI 1.07-2.02 per 30-year increment in age). The credible intervals for sex-treatment interactions included the null for SGLT2i and GLP1ra.

**Conclusions and Relevance:**

SGLT2i, GLP1ra, and DPP4i were associated with HbA1c lowering across age and sex groups. SGLT2i and GLP-1ra were associated with lower risk of MACE, with findings suggesting SGLT2i were more cardioprotective in older than younger people despite smaller HbA1c reductions, whereas GLP-1ra were more cardioprotective in younger individuals.

Over the past 2 decades, new glucose lowering agents have altered the management of type 2 diabetes. The efficacy of agents such as SGLT2 inhibitors (SGLT2i) and GLP1 receptor agonists (GLP1ra) in improving cardiovascular and kidney outcomes is established,^1,2^ with widespread use in clinical practice and inclusion in clinical guidelines.^3^ However, the possibility that treatment effects may differ depending on participant characteristics has led to questions about applying trial findings to individuals less represented in trials, such as older people and women.^4–6^

Global estimates indicate that 1 in 5 people aged over 65 years live with diabetes^10^ and that almost half of those with type 2 diabetes are aged over 65 years.^8,11^ Moreover, age-related functional limitations and conditions such as frailty typically manifest earlier in people with type 2 diabetes.^12^ The risk of complications of diabetes increases with age, potentially increasing the absolute benefits of treatment. Conversely, older adults may also be more susceptible to hypoglycemia with intensive glycemic targets.^13,14^ Among females, absolute risk of type 2 diabetes and cardiovascular disease are lower than in males, but diabetes is associated with a greater relative increase in cardiovascular risk in females than males.^15,16^ Female patients also have different patterns of cardiovascular complications and less intensive management of cardiovascular risk factors than male patients.^17^ It is therefore important to determine whether treatment effects differ by age and sex.^7–9^

Clinical guidelines do not currently recommend different diabetes therapies for male and female patients, nor across different age groups. They have, however, highlighted the uncertainty that comes from the under-representation of female participants and older people within trials.^3,18^ We aimed to perform a systematic review and meta-analysis of both aggregate and individual participant trial data to estimate whether the efficacy of SGL2i, GLP1ra and DPP4i therapy for type 2 diabetes differs by age and sex.

## Methods

This systematic review and network meta-analysis followed a prespecified protocol (PROSPERO:CRD42020184174).^22^ The protocol covers a wider project for calibration of the network meta-analysis to a community sample, seeking to provide estimates of efficacy reflecting representative samples. This manuscript presents findings from the assessment of age- and sex-treatment interactions prior to calibration. Findings are reported according to Preferred Reporting In Systematic Reviews and Meta-analyses (PRISMA) guidelines.^23^

### Eligibility criteria and search strategy

Eligible studies were randomized trials that enrolled adults greater than or equal to 18 years of age diagnosed with type 2 diabetes and assessed efficacy of SGLT2i, GLP1ra, or DPP4 inhibitors (DPP4i) on either glycated hemoglobin (HbA1c) or major adverse cardiovascular events (MACE, defined as death from cardiovascular causes, non-fatal myocardial infarction or non-fatal stroke) compared with either placebo or an active comparator of any other drug class. We excluded within-class comparisons and trials that were not registered. We included trials regardless of whether they assessed superiority or non-inferiority. For trials with cross-over designs, we included only data before the cross-over.

We searched 2 electronic databases (Medline and Embase) using both keywords and Medical Subject Headings (full search terms shown in the Supplement) as well as the US and Chinese clinical trial registries from inception to November 2022. All titles and abstracts were screened, retaining all potentially eligible studies for full text review. All stages of screening were completed by 2 reviewers working independently, with conflicts resolved by consensus and involving a third reviewer where required. In August 2024 we updated our search to include results of identified eligible registered trials published after the initial search date.

For all eligible trials, we assessed whether individual participant data were available for analysis by third party researchers through the Vivli repository and applied to the independent steering committee for access.

### Data extraction

Drug names, doses and regimens were extracted from text strings obtained from clinicaltrials.gov and published documents (papers and clinical study reports). Age and sex at baseline were obtained from published documents for aggregate trials or from the individual participant data. HbA1c results were extracted from clinicaltrials.gov or published documents. For trials with individual participant data, HbA1c values at baseline and at the time of the primary endpoint were extracted. Where endpoint values were missing, the last available observation was carried forward. As a sensitivity analysis, the baseline observation was carried forward. For MACE, results were obtained via manual extraction from published documents (including age- and sex-subgroups). MACE was defined as cardiovascular death, non-fatal myocardial infarction, or non-fatal stroke (3-point MACE). For trials with individual participant data, this definition was harmonized across trials using adjudicated events. For the aggregate data, findings for 3-point MACE were extracted to allow consistent comparison across studies. Individual-level trial data were cleaned and harmonized in the Vivli repository.

Data on adverse events were also extracted from the individual participant data, focusing on serious adverse events and events with established associations with each drug class. For each trial, incident serious adverse events, gastrointestinal adverse events, urinary tract infections, hypoglycemic episodes, amputations, and ketoacidosis were identified. Adverse events were not assessed in the aggregate trials due to a lack of harmonized definitions. Risk of bias was assessed in each study using the Cochrane Risk of Bias tool.^24^

### Statistical analysis

Detailed description of the statistical analysis is in the eMethods (Supplement). First, the age- and sex-distribution were summarized for each trial using IPD, where available, or from published summary statistics. Then, multilevel network meta-regression models were fitted for HbA1c and MACE using the multinma package in R,^25^ as previously described.^22,25^ This modelling approach was chosen as it does not disrupt randomization, makes less stringent assumptions than standard network meta-analysis, and can (without causing aggregation bias) accommodate individual participant data, aggregate-level trial data and subgroup-level trial data in models estimating treatment-covariate interactions. For HbA1c network meta-analyses were separately fit for trials of mono-, dual- and triple-therapy, reflecting different indications for the drugs in question. All MACE trials were analyzed together as their participants were selected based on cardiovascular risk. Treatment groups evaluating the combined effect of 2 or more treatments were excluded. For SGLT2i, GLP1ra, DPP4i, and metformin, treatment groups were categorized by drug and dose. Insulin was modelled as a single category. For the remaining drug classes, groups within the same trial with different doses but the same drug were combined into a single group. For all models, placebo was the reference treatment.

Trial-level regression models of each outcome by age, sex and treatment were fitted for trials with individual participant data, and age-treatment and sex-treatment interactions were assessed. Linear regression models were fitted for HbA1c that included HbA1c at baseline as a covariate. The last recorded value was carried forward in participants who did not complete the trial. Cox regression models were fitted for the MACE outcomes. Non-cardiovascular death was treated as a competing event in analyses of MACE outcomes, and cause-specific hazard ratios are presented. Cause-specific hazard ratios for the competing event were also estimated for non-cardiovascular mortality (defined where death occurred prior to first MACE). Proportional hazards assumptions were checked in the Cox models by plotting scaled Schofield residuals. Residual plots and restricted cubic splines of age were inspected for non-linearity for HbA1c and MACE outcomes. Individual participant data estimates were meta-analyzed along with aggregate trial-level and (for MACE) subgroup-level data on trial outcomes and on the age- and sex-distributions of each trial. For adverse event data, quasipoisson and negative binomial regression models were fitted for incident events within the individual participant data and meta-analyzed the results. Placebo was used as the reference category. Models were summarized using the posterior mean and 95% credible interval for the main effect and age-treatment and sex-treatment interactions. The 95% credible intervals indicate a plausible range of values; hence, when the 95% credible interval includes the null (zero for the HbA1c comparisons and 1 for the MACE comparisons) “no effect” or “no interaction” is among the plausible interpretations. To allow comparisons across the outcomes, we repeated the main analyses restricting the data to the 14 trials with individual-level or aggregate data for both HbA1c and MACE. None of the analyses employed formal adjustment for multiple testing. Individual participant data summaries and aggregate level data are available at the project github repository https://github.com/Type2DiabetesSystematicReview/nma_agesex_public.

## Results

### Systematic review results

**Figure 1:**
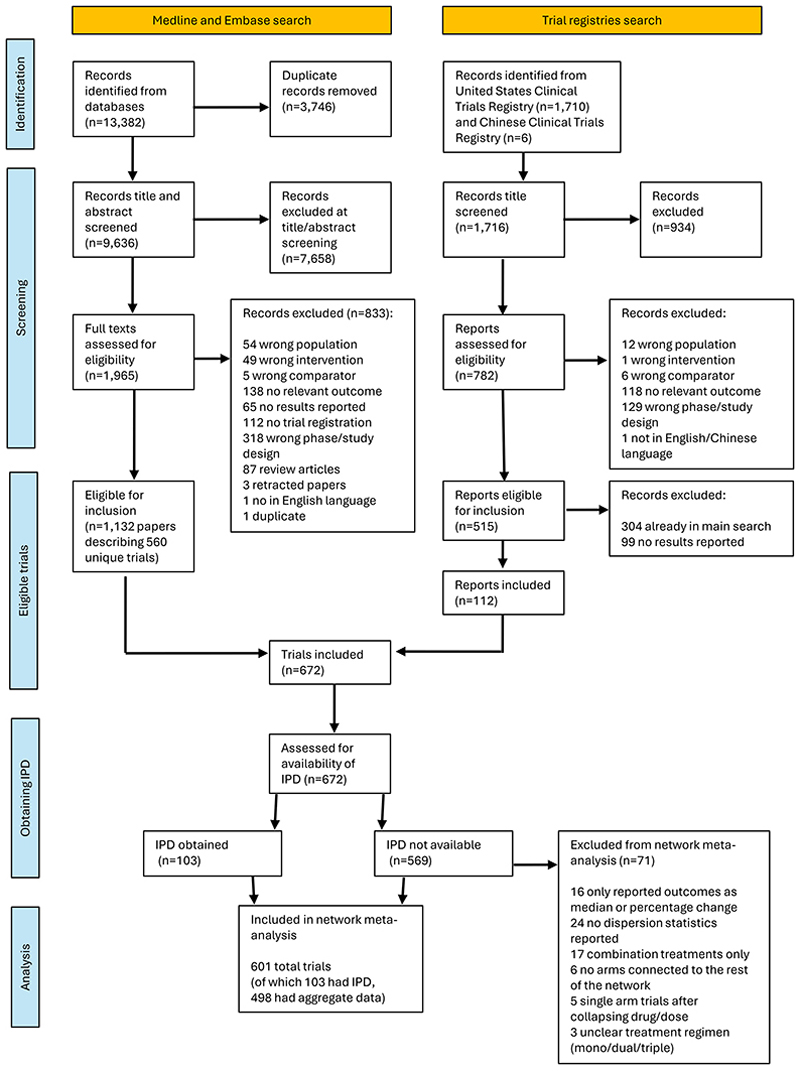
Identification and Accrual of Included Trials This figure shows the screening and selection of eligible trials and the subsequent acquisition of IPD (individual participant data). Trials without results in English/Chinese were excluded due to a lack of available translation.

We identified 687 eligible trials and included 601 in the network meta-analyses (Figure 1). Of these, 592 reported HbA1c outcomes, 23 reported MACE outcomes, and 14 reported both. A total of 498 aggregate level trials included 303,311 participants, and 103 individual participant data trials included 92,182 participants. Trial-level details and risk of bias are shown in the online project repository.

Table 1 shows the total number of included trials reporting HbA1c for each drug class along with aggregate baseline characteristics. Characteristics were similar for trials with individual participant data and those with aggregate data. For trials reporting MACE, trial-level details are shown in Table 2. There were more male than female participants, and the age range of almost all trial participants was 40 to 80 years, including trials targeted at older people (eFigure1, eTable1, Supplement).

### Main treatment effects

The main treatment effects for HbA1c comparing each treatment versus placebo are shown for a standard network meta-analysis without covariates in eFigure 2. Treatments reduced HbA1c with a range of absolute reductions of -0.5% to -1.5%. The main treatment effects for MACE show a reduced hazard of MACE for SGLT2i and GLP1ra compared with placebo, with null findings for DPP4i (eFigure3).

### Age-treatment and sex-treatment interactions

**Figure 2:**
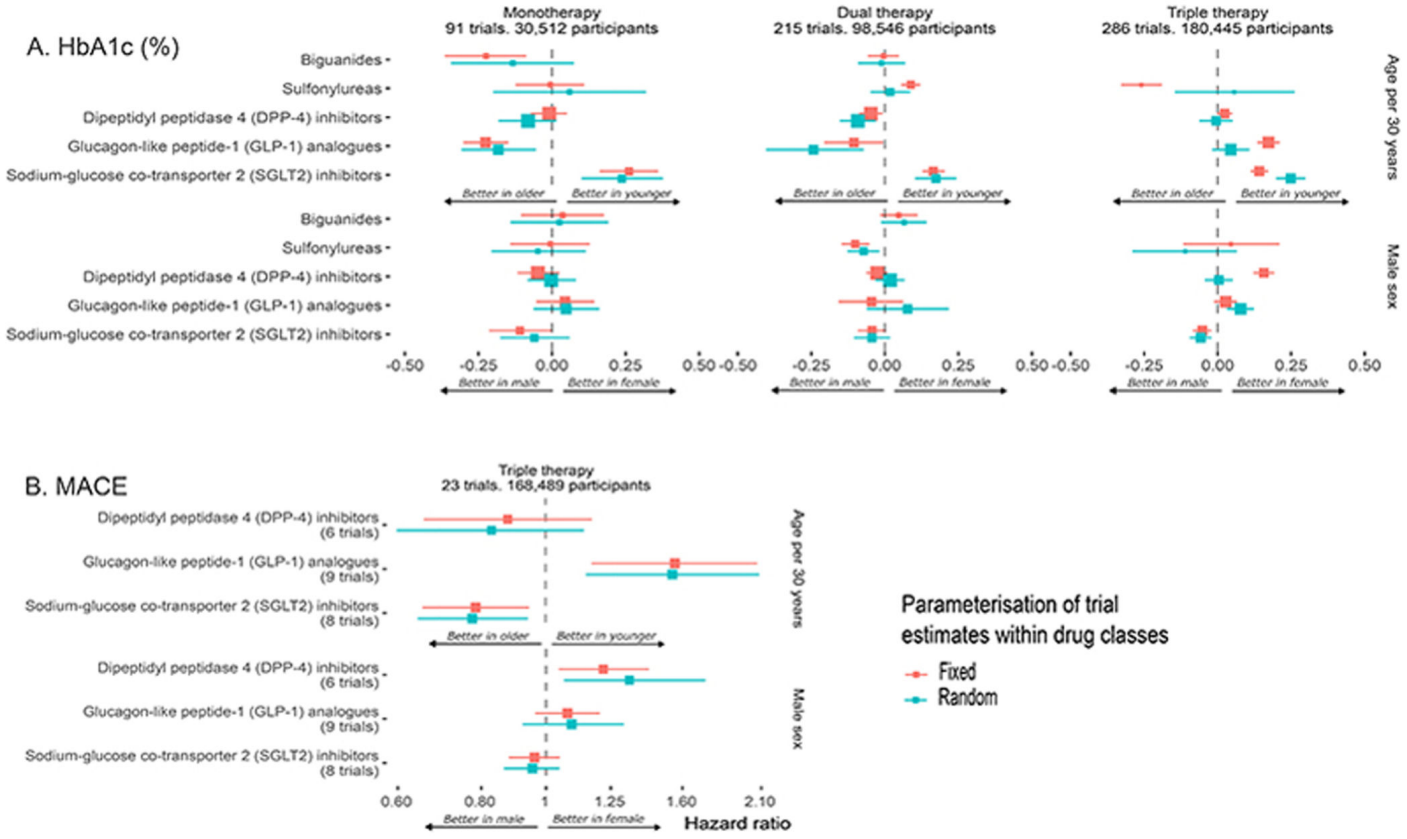
Covariate-treatment interactions for HbA1c and MACE This figure shows the covariate-treatment interaction estimates for age and sex represented as dots, both for a) HbA1c (top pane3ls) and b) MACE (bottom panel). Horizontal lines show the 95% credible interval. Age was modeled as a continuous variable and divided by 30 (so that the coefficient reflects the difference in efficacy over a 30-year age difference). Estimates below the line of no effect (dashed vertical line) indicate that the treatment is more efficacious in older age/in male sex. Estimates above this line indicate the inverse. The area of each point represents the proportion of participants in the analysis who had been allocated to a drug in that class. Mono-, dual and triple therapy indicates trials where, in addition to the study drug participants are required or permitted to also be taking no other, one additional other or two or more additional other antidiabetic medications. The fixed and random effects refer to the main treatment effects (eg canagliflozin 300 mg).

Figure 2 shows age-treatment and sex-treatment interactions, assessing differences in the efficacy of treatment by age and sex, for HbA1c and MACE. SGLT2-inhibitors had less absolute HbA1c lowering with increasing age (0.24%; 95% CrI 0.10-0.38, 0.17%; 95% CrI 0.10-0.24 and 0.25%; 95% CrI 0.20-0.30 less HbA1c lowering per 30-year higher age for monotherapy, dual therapy, and triple therapy, respectively). There was no evidence for non-linearity in the age-treatment interaction (eFigure 4). Results were also similar confining the analysis to trials with greater than or equal to 6 months of follow-up (eFigure5). GLP1ra had greater absolute effects on HbA1c lowering with increasing age in monotherapy and dual-therapy (-0.18%; 95% CrI -0.31 to -0.05 and -0.24%; 95% CrI -0.40 to -0.07 HbA1c lowering per 30-yer increment respectively) but not triple therapy trials (0.04%; 95% CrI -0.02-0.11 per 30-year increment). DPP-4i had slightly better absolute HbA1c lowering in dual-therapy for older people (-0.09%; 95% CrI -0.15 to -0.03 HbA1c lowering per 30-year increment), but no evidence of variation in efficacy for mono or triple therapy (-0.08%; 95% CrI -0.18 to 0.01 and -0.01%; 95% CrI -0.06 to 0.05 HbA1c lowering per 30-year increment respectively). There was no variation in efficacy by sex except for a small difference in efficacy of SGLT2i favoring males for triple therapy only (-0.06%; 95% CrI -0.18 to 0.06).

Older people had greater relative reduction in MACE for SGLT-2i (HR 0.76; 95% CrI 0.62-0.93 per 30-year increment in age) and less relative reduction in MACE for GLP1ra (HR 1.47; 95% CrI 1.07-2.02 per 30-year increment in age), with the credible interval for DPP-4i including the null (HR 0.73; 95% CrI 0.52-1.00). When modeling sex-treatment interactions in MACE trials, DPP-4i were less efficacious in male participants (HR 1.65; 95% CrI 1.25-2.21 for male versus female), although this association was attenuated after including sex-subgroup data in the analysis (HR 1.22; 95% CrI 1.04-1.42) and after excluding the only DPP-4i trial with individual participant data the credible interval included the null (eFigure 6). For GLP1ra (HR 1.17; 95% CrI 0.87-1.58 for male versus female) and SGLT-2i (HR 0.95; 95% CrI 0.86-1.06 for male versus female), there was no evidence for a sex-treatment interaction. Additional models did not show non-linearity of the age-treatment interaction within the range of ages included in the trials (eFigure7).

Sensitivity analyses including or excluding age- and sex-subgroup data in the model did not affect HbA1c findings in older people taking SGLT2i, except for an analysis excluding 1 of the 4 SGLT2i trials with individual participant data (eFigure6). The greater relative reduction in MACE risk at older ages was preserved or greater in all sensitivity analyses. Similar results were obtained in analyses restricting the data to the 14 trials with individual-level data for both HbA1c and MACE (eFigure8). Results of MACE analyses differed depending on the inclusion or exclusion of single trials of GLP1ra and DPP-4i with individual participant data and the inclusion or exclusion of subgroup data (eFigure6).

There was no age- or sex-treatment interaction between any class of medication and gastrointestinal adverse events, hypoglycemia, or urinary tract infections (eFigure9). There were no age- or sex-treatment interactions with serious adverse events for SGLT-2i, GPP-1ra, or DPP4i (eFigure9). Death was uncommon across trials (eFigure10), and there was no evidence for any age-treatment or sex-treatment interactions for non-cardiovascular death (eFigure11). There were too few events within the individual participant trial data to fit models for amputation or ketoacidosis (eTable2).

### Age and sex-specific effects for MACE trials

**Figure 3:**
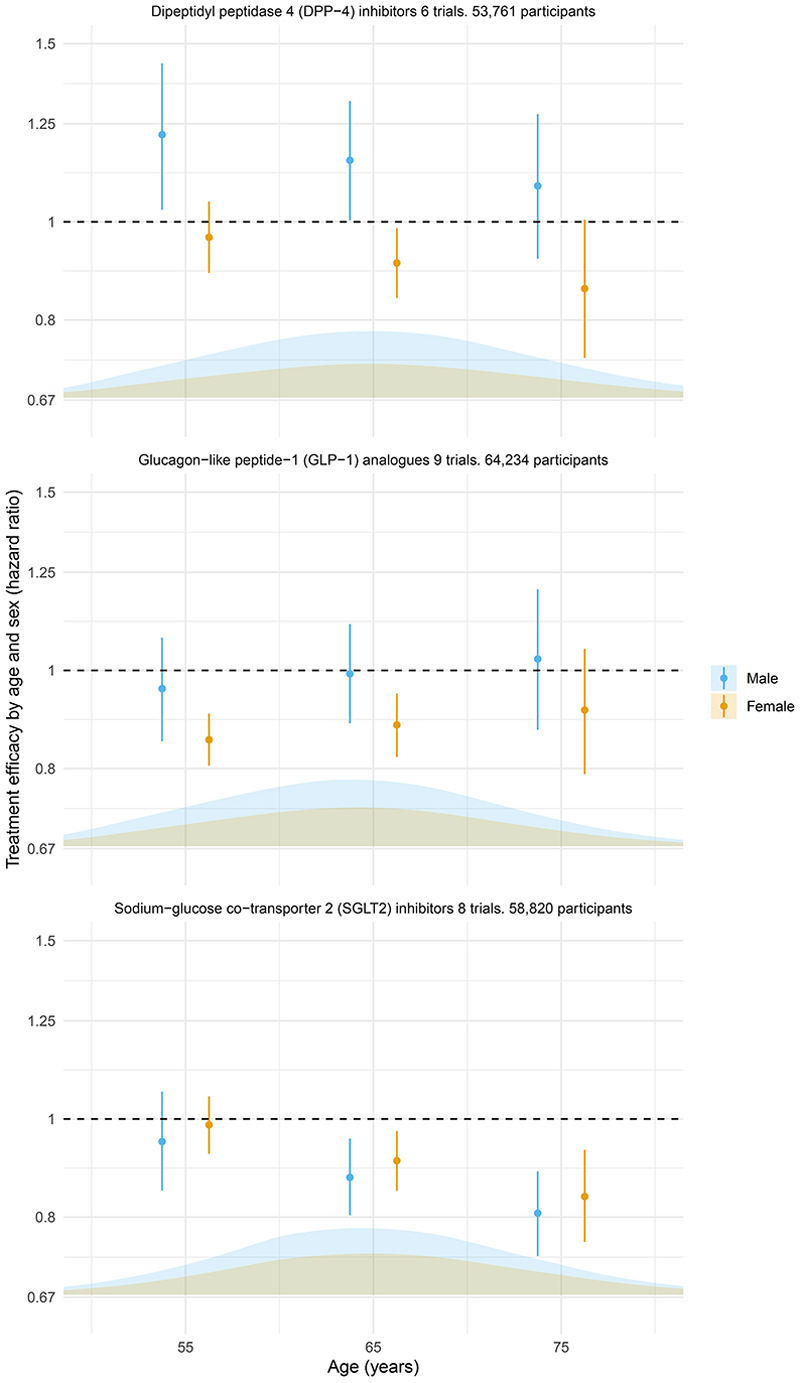
Relative effects for MACE This figure is based on a model including all available trials, including sex-subgroup data as well as aggregate data and individual participant data. Points and line-ranges show age- and sex-specific estimates of the effect of each treatment compared to placebo on the hazard of MACE. The density plots indicate the proportion of trial participants of by sex and across the age ranges.

Figure 3 shows associations between age-treatment and sex-treatment interactions and the overall age- and sex-specific relative efficacy versus placebo for each class. SGLT2i were associated with reduced MACE in older people regardless of sex (HR 0.84; 95% CrI 0.76-0.93 for 75-year old females and 0.81; 95% CrI 0.73-0.89 for 75-year old males and 0.91; 95% CrI 0.85-0.97 for 65-year old females and 0.88; 95% CrI 0.80-0.96 for 65-year old males). For GLP1ra, there was no association with a significant reduction in MACE in male participants (eg HR 0.99; 95% CrI 0.89-1.11 in 65 year old males) and in older people (HR 0.91; 95% CrI 0.79-1.05 for 75 year old females and 1.03; 95% CrI 0.87-1.20 for 75-year old males), but there was a decreased risk of MACE in younger female participants (HR 0.85; 95% CrI 0.81-0.91 in 55 year old females and 0.88; 95% CrI 0.82-0.95 in 65 year old females). These findings should be interpreted with caution. Although the GLP1ra class showed an overall benefit for MACE (eFigure 3), the effect on MACE for some of the drugs within this class was null (eFigure 12). Similarly, while there were some differences in efficacy across age and sex for DPP4i, these should be interpreted with caution since these agents showed a null overall effect on MACE. All interaction estimates were sensitive to the inclusion of specific trials.

eTable2 in the Supplement provides heterogeneity estimates for all of the random effects models.

## Discussion

This network meta-analysis of 601 trials, including IPD from 103 trials, assessed whether the efficacy of three newer drug classes (SGLT2i, GLP1ra and DPP4i) varied by age or sex in people with type 2 diabetes. For HbA1c, SGLT2i showed modestly reduced efficacy with increasing age, with attenuation of the treatment effect compared to placebo by approximately 0.25% at 75 compared with 45 years of age. In contrast, the reduction in MACE with SGLT2i was greater in older compared to younger people. For GLP1ra there was some evidence that HbA1c lowering was greater in older individuals, whereas cardiovascular efficacy was greater among younger female participants.

Previous studies assessing heterogeneity in efficacy, that is, interaction, of type 2 diabetes treatment by age or sex have generally used aggregate or subgroup data from randomized controlled trials, or relied on observational (i.e., non-randomized) data. A meta-analysis of differences between male and female participants in the efficacy of SGLT2i and GLP1ra found no statistically significant difference in efficacy for cardiovascular outcomes but speculated on possible reduced cardiovascular efficacy among female patients due to the greater statistical uncertainty in the estimates for this group.^7^ Our analysis, including a larger and more comprehensive group of studies and incorporating individual participant data, provides greater precision and more clearly demonstrated that sex is not associated with any difference in the efficacy of these classes of medication.

A recent network meta-analysis assessed the efficacy of type 2 diabetes treatment across a range of clinical outcomes, including heart failure, end-stage kidney disease, and medication related-harms not included in the present analysis.^2^ This recent network meta-analysis showed that, in addition to MACE, SGLT2i and GLP1ra reduced the risk of admission to hospital with heart failure and the risk of end-stage kidney disease, with superior efficacy of SGLT2i in reducing end-stage kidney disease. Harms with treatment were generally class-specific and included genital infections with SGLT2i and gastrointestinal complications with GLP1ra. This previous analysis, however, did not assess heterogeneity by age and sex, and did not include analysis of IPD.

One likely explanation for the reduction in glycaemic efficacy of SGLT2i with older age is age-related decline in kidney function. For example, a recent double-blind 3-way crossover study comparing DPP4i with SGLT2i demonstrated that participants with estimated glomerular filtration rates 60-90 ml/min/1.73m^2^, compared with those >90 ml/min/1.73m^2^, had lower HbA1c while taking DPP4 inhibitors than while taking SGLT2 inhibitors.^26^ In this context, it is notable that the reductions in MACE with SGLT2i were greater in older people, despite lower glycemic efficacy. This highlights the limitation of surrogate outcomes such as HbA1c in determining the risks of MACE, for which hyperglycemia is a less important risk factor than hypertension or dyslipidemia.^27^ It is also consistent with the established efficacy of SGLT2 inhibitors for improving cardiovascular outcomes in conditions other than diabetes, such as heart failure or chronic kidney disease, which are not characterized by hyperglycemia. Current clinical guidelines recommend less stringent glycemic targets in older people living with multiple long-term conditions or frailty due to greater risks of adverse events.^3,28^ The current findings highlight the need to consider cardioprotective effects of therapies, in addition to safety, tolerability and patient’s priorities, when treating older people. While our findings demonstrate similar or better cardiovascular efficacy among older people within the included trials, trials rarely enroll people over 80 years of age. There are also likely to be unmeasured differences between trial participants and people considered for treatment in routine care. For example, age-associated states such as frailty, which increase the risk of both cardiovascular events and complications,^13,29^ are not quantified in these trials.^30^ This analysis does not, therefore, assess whether efficacy is similar in people of much higher ages (i.e. over 80 years) or living with frailty. This is a group in which the balance of risks and benefits is most uncertain. Moreover, it is likely that the effect of age on treatment efficacy is moderated through other measurable age-related characteristics such as kidney function or the presence and extent of comorbidities. Accounting for such characteristics in future work may allow more nuanced understanding of the likely benefits of treatments according to more specific characteristics, determining not only the overall treatment efficacy in older people (for example) but in older people with different physiological and clinical characteristics.

There is a need for trials that recruit and retain older people and those living with frailty, and which explicitly measure and report functional status.

### Limitations

First, while the primary strength of this analysis is in the use of individual participant data to estimate age- and sex-treatment interactions, this was not available for all included trials. Individual participant data improves statistical power and allows integration of individual participant data and aggregate data within network meta-analysis to preserve randomization and avoid aggregation bias. We also followed rigorous systematic review methodology to identify eligible studies and have made all model outputs and analysis code publicly available to facilitate replication of our findings. However, despite the inclusion of a large volume of individual participant data, it was not available for all trials (103/601, 17%).

Furthermore, the trials for which we did have individual participant data were not a random sample of the included trials as their availability depended on the sponsor’s data sharing arrangements. We did not attempt to obtain additional individual participant data through direct contact with study authors. Second, our use of multi-level network meta-regression also meant that all treatment comparisons -within class, between class, and versus placebo – whether or not individual-level data was available – could be used to estimate the interactions. Treatment effects within classes were estimated independently; drugs within a class were *not* assumed to have the same or similar efficacy. However, to estimate the interactions from the available data, our approach assumes that interactions are common across drugs in the same class, and in practice it also requires at least some trials with individual-level data for each class. Third, while we included a large number of trials, a relatively small proportion of these assessed cardiovascular outcomes. Fourth, we dropped trial groups with multiple drug classes as the software does not allow for explicit modeling of components within groups, and our focus was on class-level interactions. Fifth, while we assessed glycemic and cardiovascular efficacy, which are clinically relevant outcomes, our analysis did not include other clinical endpoints (such as kidney events). Sixth, while we assessed whether the association between these medications and established risks varied by age and sex, these analyses were limited by the small number of events within the trial data. Furthermore, we did not attempt to identify novel associations between these agents and specific adverse events. Such analyses would ideally draw on both trial data and routine healthcare data, in which identification of rarer events is more feasible. Seventh, we did not present MACE in terms of absolute risks. In most settings, it is likely that MACE is higher with age, which would tend to increase the absolute benefits of treatment. However, competing risks (e.g., non-cardiovascular mortality) are also likely to be higher with age.

Consequently, the absolute benefit of treatment in older people will depend not only on the relative treatment effects, but also on the rates of MACE and competing events in the target population.

## Conclusions

SGLT2i, GLP1ra, and DPP4i were associated with HbA1c lowering across age and sex groups. SGLT2i and GLP-1ra were associated with lower risk of MACE, with findings suggesting SGLT2i were more cardioprotective in older than younger people despite smaller HbA1c reductions, whereas GLP-1ra were more cardioprotective in younger individuals.

## Supplementary Material

Supplementary appendix

## Figures and Tables

**Table 1:** Trials reporting HbA1c, comparisons and characteristics

Classes	Dipeptidyl peptidase 4 inhibitors		Glucagon-like peptide-1 analogues		Sodium-glucose co-transporter 2 inhibitors		Total trials	
	Aggregate	IPD	Aggregate	IPD	Aggregate	IPD	Aggregate	IPD
Total	237	43	158	34	140	32	489	103
Placebo	120	31	68	20	95	21	278	72
**Specific drugs of the following classes ^a^**								
Dipeptidyl peptidase 4 (DPP-4) inhibitors	-	-	19	3	18	6	266	52
Glucagon-like peptide-1 (GLP-1) analogues	26	3	-	-	9	0	223	49
Sodium-glucose co-transporter 2 (SGLT2) inhibitors	19	9	9	0	-	-	175	55
Sulfonylureas	26	4	8	1	12	3	45	7
Biguanides (metformin only)	23	9	4	1	3	3	29	13
Thiazolidinediones	15	0	5	1	4	0	22	1
Alpha glucosidase inhibitors	12	1	2	0	1	0	14	1
‘Other blood glucose lowering drugs, excl. insulins’, eg repaglinide	2	0	0	0	0	0	2	0
**Any drug of the following class**								
Insulins and analogues (eg “any insulin”)	5	0	40	8	1	0	44	8
Blood glucose lowering drugs, excl. insulins (eg “any oral antidiabetic drug”)	1	0	3	0	0	0	4	0
								
2 groups ^b^	204	27	106	22	107	12	388	56
3 groups ^b^	25	10	41	9	28	16	80	34
4 or 5 groups ^b^	8	6	11	3	5	4	21	13
Participants	109293	29991	79184	28137	44039	40191	217321	92182
Male n (%)	63066 (57.7%)	16724 (55.8%)	44780 (56.6%)	16309 (58.0%)	24776 (56.3%)	24638 (61.3%)	124159 (57.1%)	54465 (59.1%)
Female n (%)	46227 (42.3%)	13267 (44.2%)	34404 (43.4%)	11828 (42.0%)	19263 (43.7%)	15553 (38.7%)	93162 (42.9%)	37717 (40.9%)
Age, years (sd) [5^th^ to 95^th^ centile]	58.8 (10.8) [40.2-75.8]	57.2 (11.2) [36.9-75.1]	57.9 (10.3) [40.3-74.2]	59.3 (11.0) [40.0-76.1]	61.3 (10.7) [43.1-78.1]	57.8 (11.2) [36.4-75.2]	59.1 (10.7) [40.9-76.0]	58.3 (11.2) [37.6-75.6]
Duration, weeks median (5^th^ to 95^th^ centile)	24.0 (12.0- 54.4)	24.0 (12.2- 53.8)	26.0 (12.0- 56.0)	26.0 (24.0- 52.0)	24.0 (12.0- 52.0)	25.0 (17.1- 239.2)	24.0 (12.0- 56.0)	24.0 (14.2- 104.0)

a The number of trials in each class do not sum to the total because some trials include more than one class. Trials may contribute data to more than one cell in this table (e.g. where a trial compares two different classes of glucose-lowering agents in separate groups, this trial would contribute to the total of each of these classes within this table).

b Groups refers to the number of comparisons within the trial, after collapsing groups comparing different doses of the same agents.

**Table 2:** MACE Trials, characteristics

(a) Asterisk indicates trial without a placebo group. AGG aggregate level data only, SG subgroup level data only, IPD IPD available.							
Class	Trial	Data level	Treatment	Participants	Follow-up (years)	Male (%)	Age, years mean(SD)[5-95th centile]
Dipeptidyl peptidase 4 DPP-4inhibitors	TECOS NCT007902 05	AG G	sitagliptin 100 milligram	14671	5.0	70.7	65.6 (8.0) [53.2-79.4]
	SAVOR- TIMI-53 NCT011078 86	SG	saxagliptin 5 milligram	16492	2.9	66.9	65.2 (8.5) [51.1-79.1]
	CAROLINA NCT012434 24	SG	glimepiride 1 milligram vs linagliptin 5 milligram*	6033	8.3	60.0	64.0 (9.7) [47.2-80.1]
	NCT017032 08	AG G	omarigliptin 25 milligram	4202	3.4	70.2	63.6 (8.6) [49.8-77.7]
	CARMELIN A NCT018975 32	SG	linagliptin 5 milligram	6979	4.3	62.9	65.8 (9.0) [50.9-80.5]
	EXAMINE NCT009687 08	IPD	alogliptin 25 milligram	5384	3.3	67.9	60.8 (9.9) [44.6-77.2]
Glucagon -like peptide-1 receptor GLP-1analogues	EXSCEL NCT011443 38	SG	exenatide 2 milligram	14752	7.5	62.0	61.7 (9.5) [46.3-77.3]
	ELIXA NCT011472 50	AG G	lixisenatide 20 microgram	6068	3.9	69.3	60.1 (9.7) [44.0-75.9]
	LEADER NCT011790 48	SG	liraglutide 1.8 milligram	9340	5.0	64.2	64.3 (7.2) [52.9-76.8]
	REWIND NCT013949 52	SG	dulaglutide 1.5 milligram	9901	8.0	53.7	66.2 (6.6) [55.4-77.3]
	FREEDOM CVO NCT014558 96	AG G	itca650 60 microgram	4156	2.0	63.3	63.0 (7.7) [50.2-75.8]
	SUSTAIN 6 NCT017204 46	AG G	semaglutide 0.5/1 milligram	3297	2.1	60.7	64.8 (7.2) [53.4-77.2]
	PIONEER 6 NCT026927 16	SG	semaglutide 14 milligram	3183	1.6	68.4	65.9 (6.9) [54.6-77.7]
	AMPLITUDE -O NCT034962 98	SG	efpeglenatide 4_6 NA	4076	2.6	67.0	64.5 (8.1) [51.0-78.0]
	HARMONY NCT024655 15	IPD	albiglutide 30 milligram	9461	2.7	69.4	64.0 (8.7) [49.7-78.3]
Sodium-glucose co-transporter 2 inhibitors	DECLARE-TIMI58 NCT017305 34	SG	dapagliflozin 10 milligram	17160	5.2	62.6	63.9 (6.7) [52.9-75.1]
	VERTIS CV NCT019868 81	SG	ertugliflozin 5/15 pooled milligram	8246	6.0	70.0	64.4 (8.1) [51.0-77.6]
	SCORED NCT033151 43	AG G	sotagliflozin 200 mg	10584	2.5	55.1	68.2 (8.5) [54.2-82.2]
	SOLOIST-WHF NCT035219 34	AG G	sotagliflozin 200 mg	1222	1.8	66.2	68.7 (9.1) [52.6-82.7]
	CANVAS NCT010326 29	IPD	canagliflozin 100 milligram vs canagliflozin 300 milligram	4330	8.0	66.1	60.8 (8.1) [47.4-74.0]
	EMPA-REG OUTCOME NCT011316 76	IPD	empagliflozi n 10 milligram vs empagliflozi n 25 milligram	7064	4.6	71.5	63.1 (8.7) [48.7-77.5]
	CANVAS-R NCT019897 54	IPD	canagliflozin 100 milligram	5813	3.0	62.8	62.5 (8.6) [48.6-76.6]
	CREDENCE NCT020657 91	IPD	canagliflozin 100 milligram	4401	4.6	66.1	56.4 (9.2) [45.0-75.0]

## Data Availability

Individual-level participant data was obtained through the Vivli project, subject to a data sharing agreement. Data are available on application to the data holder via Vivli’s application process. All aggregate data, as well as summary data from all analyses of individual participant data, are available at https://github.com/Type2DiabetesSystematicReview/nma_agesex_public, along with analysis code for all the analyses presented in the manuscript and Supplement. Thank-you to the MVLS Advanced Research (MARS) team (https://mars.ice.gla.ac.uk/) for providing the high performance computing resource we used to run the Bayesian multi-level network meta regression models. For the purpose of open access, the authors have applied a Creative Commons Attribution (CC BY) licence to any Author Accepted Manuscript version arising from this submission.

## Key points

### Question

Does the efficacy of sodium glucose cotransporter 2 inhibitors, glucagon-like peptide-1 receptor analogues, and dipeptidyl peptidase-4 inhibitors vary by age and sex in type 2 diabetes?

### Findings

In this systematic review and network meta-analysis of 601 eligible trials including 103 trials with individual participant data, there was a greater reduction in the risk of major adverse cardiovascular events, comparing older with younger participants taking sodium glucose cotransporter 2 inhibitors, despite smaller reductions in hemoglobin A1c. Sex was not associated with differences in efficacy for any agent.

### Meaning

Newer glucose lowering drugs were efficacious across age and sex groups. Sodium glucose cotransporter 2 inhibitors were more cardioprotective in older than younger people.
