# Supplementary material for: Age and sex differences in efficacy of treatments for type 2 diabetes: A network meta-analysis
Source: JAMA. Author manuscript; Available in PMC 2025 Feb 27. (PMC11791772; doi:10.1001/jama.2024.27402)
Supplement: Supplementary appendix [file EMS203121-supplement-Supplementary_appendix.docx]

Supplementary appendix

Table of contents

[eFigures 2](#_Toc183634273)

[eFigure 1a Age distribution for HbA1c trials 2](#_Toc183634274)

[eFigure 1b Age distribution for IPD HbA1c trials by sex 3](#_Toc183634275)

[eFigure 1c Age distribution 4](#_Toc183634276)

[eFigure 2 Main treatment effects for HbA1c outcome 5](#_Toc183634277)

[eFigure 3 Main treatment effects for MACE outcome by class 8](#_Toc183634278)

[eFigure 4 Non-linear age-treatment interaction estimates for each class for HbA1c 9](#_Toc183634279)

[eFigure 5 Various sensitivity analyses for age-treatment and sex-treatment interaction for HbA1c 12](#_Toc183634280)

[eFigure 6 Various sensitivity analyses for age-treatment and sex-treatment interaction for MACE 13](#_Toc183634281)

[eFigure 7 Non-linear age-treatment interaction estimates for MACE trials 15](#_Toc183634282)

[eFigure 8 Posterior distribution for age-treatment interaction for SGLT-2 inhibitor versus placebo for HbA1c and MACE. Main analysis includes all trials while sensitivity analysis includes only those trials with both HBA1c and MACE results 16](#_Toc183634283)

[eFigure 9a Age-treatment interactions (per 30-years) for adverse events 17](#_Toc183634284)

[eFigure 9b Sex-treatment interactions for adverse events 18](#_Toc183634285)

[eFigure 10 Histogram of mortality for IPD trials 19](#_Toc183634286)

[eFigure 11 Hazard ratios for non-cardiovascular death in MACE IPD trials 20](#_Toc183634287)

[eFigure 12 Main treatment effects for MACE 21](#_Toc183634288)

[eTables 22](#_Toc183634289)

[eTable 1 Mean age in years by sex and arm for IPD MACE trials 22](#_Toc183634290)

[eTable 2 Between-study heterogeneity standard deviation “tau” for main effects 23](#_Toc183634291)

[eTable 3 Adverse incident event counts and rates for IPD trials 24](#_Toc183634292)

[eMethods 30](#_Toc183634293)

[Search strategy 30](#_Toc183634294)

[Data extraction 30](#_Toc183634295)

[Statistical analysis 30](#_Toc183634296)

[Baseline characteristics 30](#_Toc183634297)

[Multi-level network meta-regression models (MLNR) 30](#_Toc183634298)

[Sensitivity analyses of ML-NMR findings 31](#_Toc183634299)

[Examinination of non-linearity in the age-treatment interactions HbA1c 31](#_Toc183634300)

[Examination of non-linearity in the age-treatment interactions for MACE 32](#_Toc183634301)

[eResults 32](#_Toc183634302)

[Non-linearity in the age-treatment interactions for HbA1c 32](#_Toc183634303)

[Models without the use of IPD 32](#_Toc183634304)

[Missingness 32](#_Toc183634305)

# eFigures

### eFigure 1a Age distribution for HbA1c trials


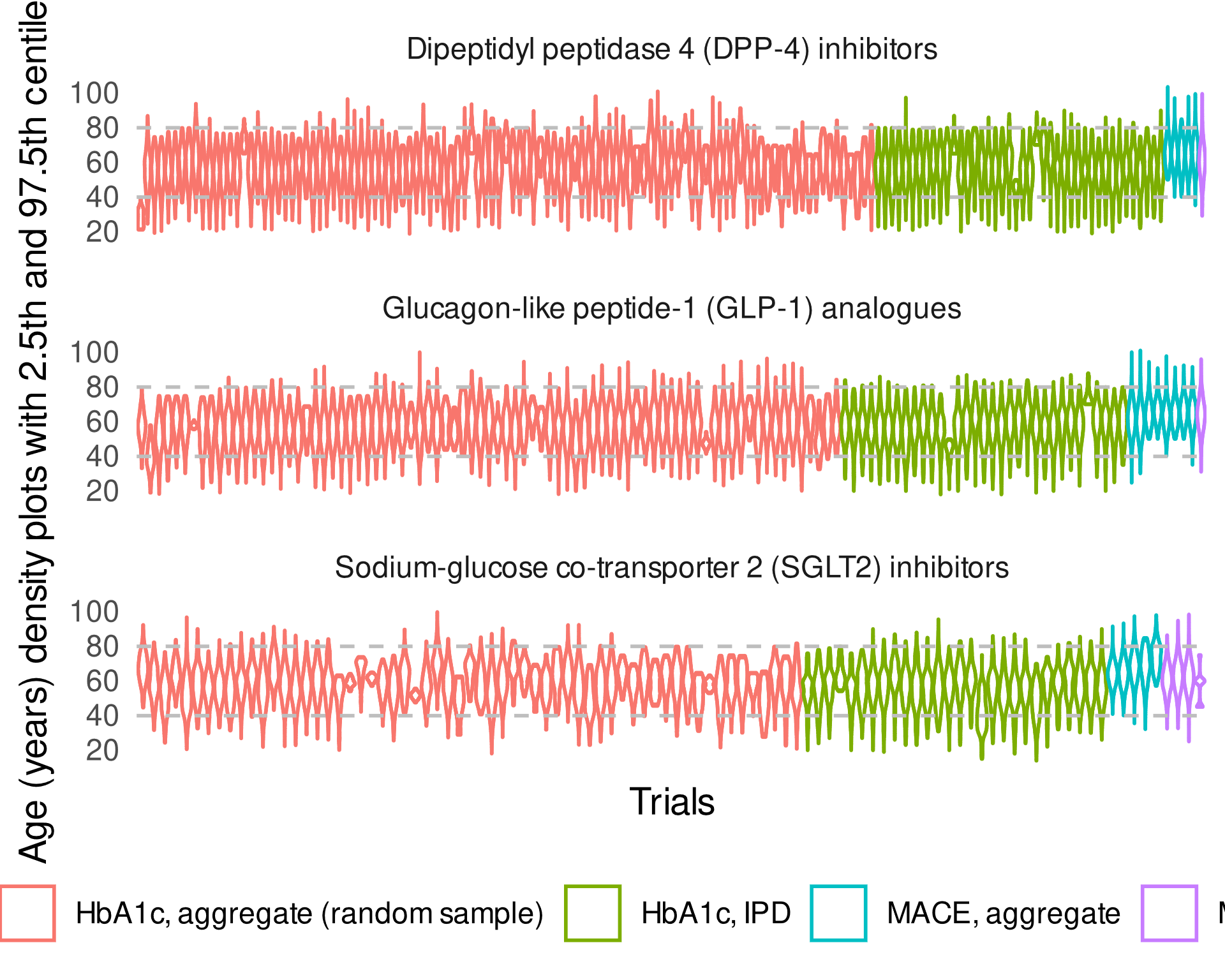


###### Age distribution shown as violin plots (similar to density plots, but turned through 90 degrees so more can be visualised). Results presented for all MACE trials, all individual-level participant data (IPD) trials and a random sample of aggregate-level HbA1c trials. Except for IPD HbA1c trials, or trials where older people were explicitly excluded, the tails of the violin plots may overestimate the presence of people over the age of 80 (as they assume no truncation). Red ink HbA1c trials with aggregate data (random sample), green ink HbA1c trials with IPD, blue ink MACE trials with aggregate data, purple ink MACE trials with IPD.

### eFigure 1b Age distribution for IPD HbA1c trials by sex


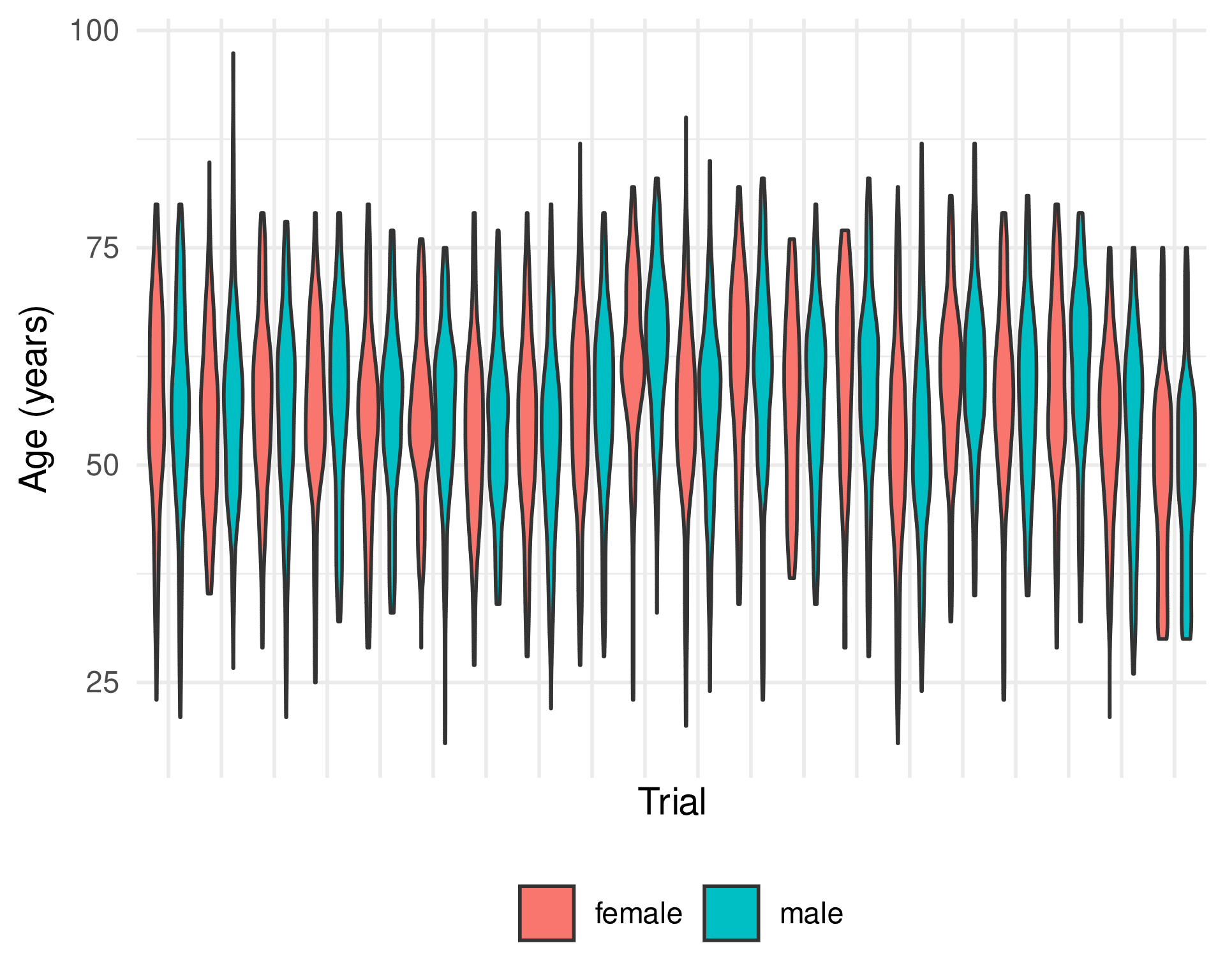


###### As eFigure 1a, stratified by sex.

### eFigure 1c Age distribution


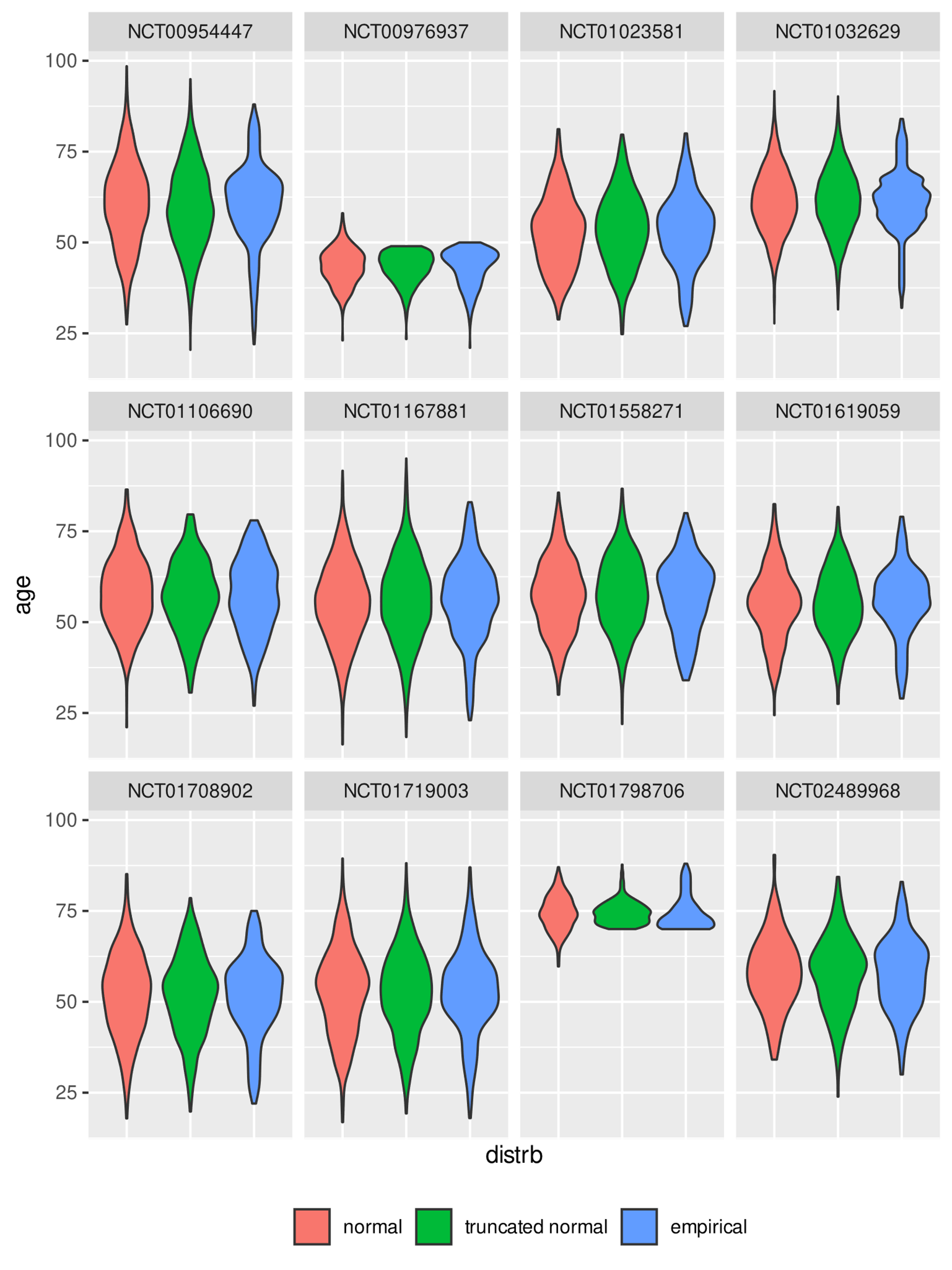


###### Plot examining extent to which the truncated normal distributions estimated from summary statistics match empirical cumulative distribution functions (ECDFs). Random sample of trials shown here. Figure with all trials is available at <https://github.com/Type2DiabetesSystematicReview/nma_agesex_public/tree/main/Outputs/ef1.pdf> and density plots (ie not violin plots) at <https://github.com/Type2DiabetesSystematicReview/nma_agesex_public/tree/main/Outputs/age_plots_check_simulation_density_plots.pdf>

### eFigure 2 Main treatment effects for HbA1c outcome


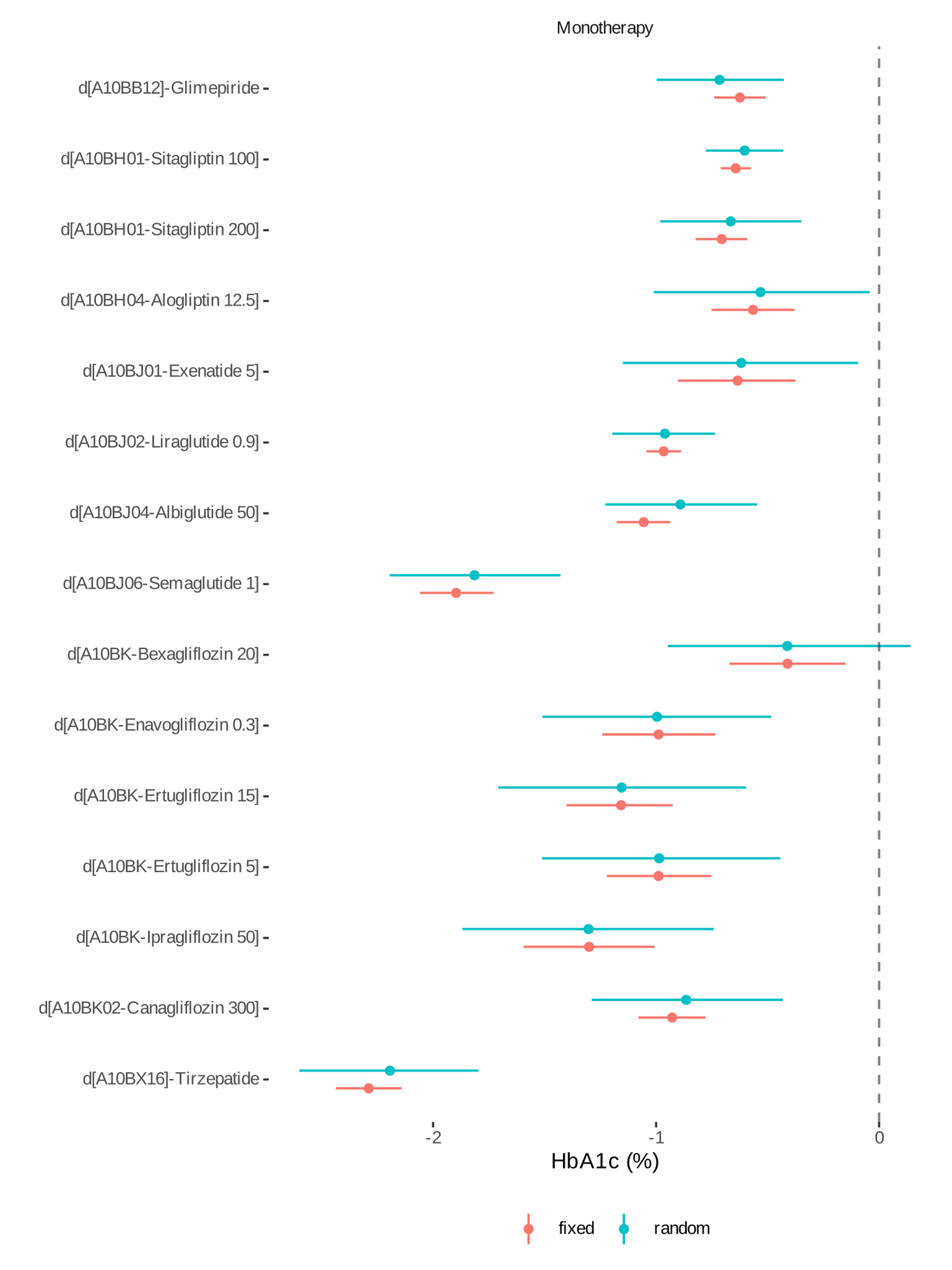


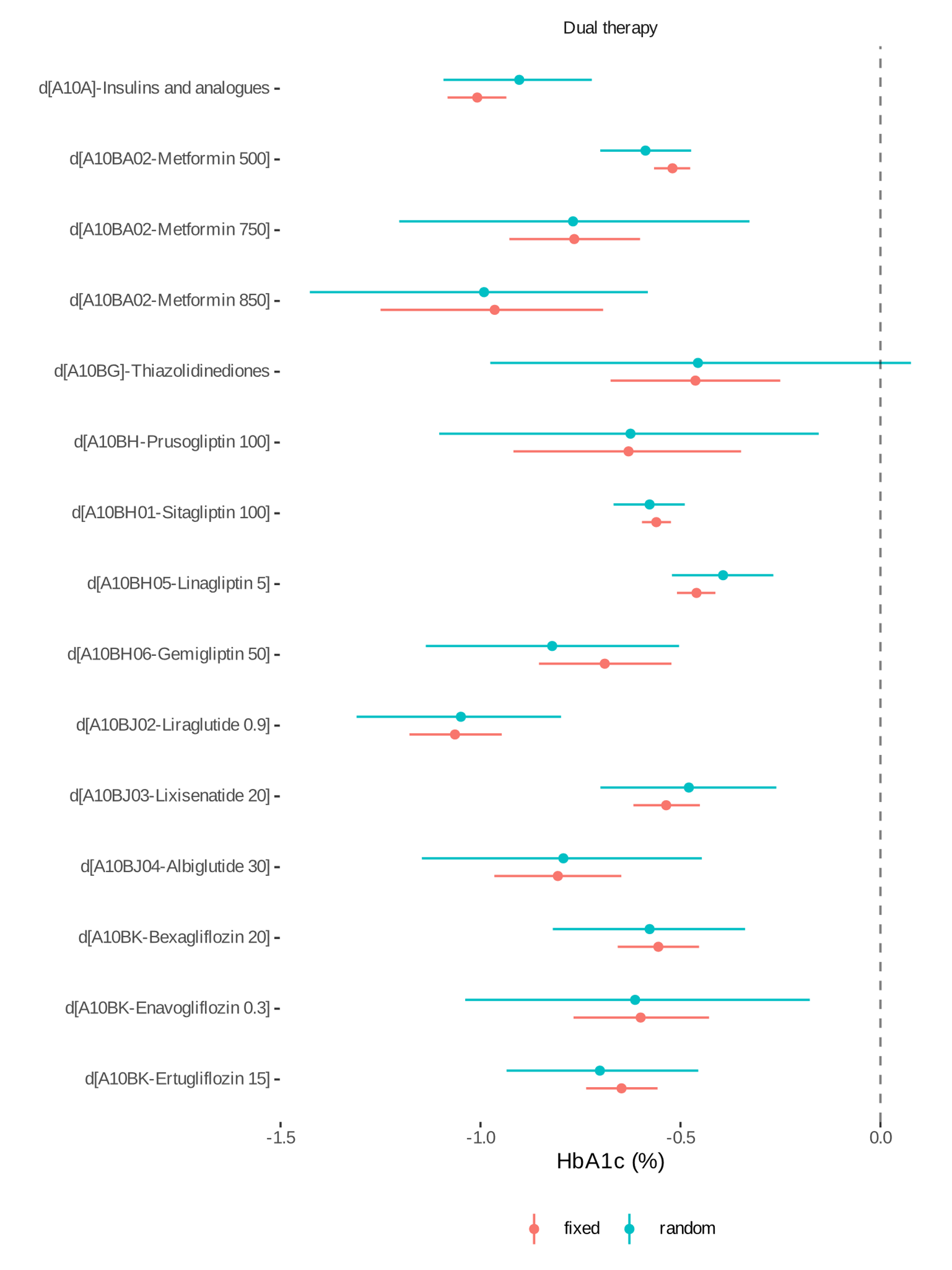


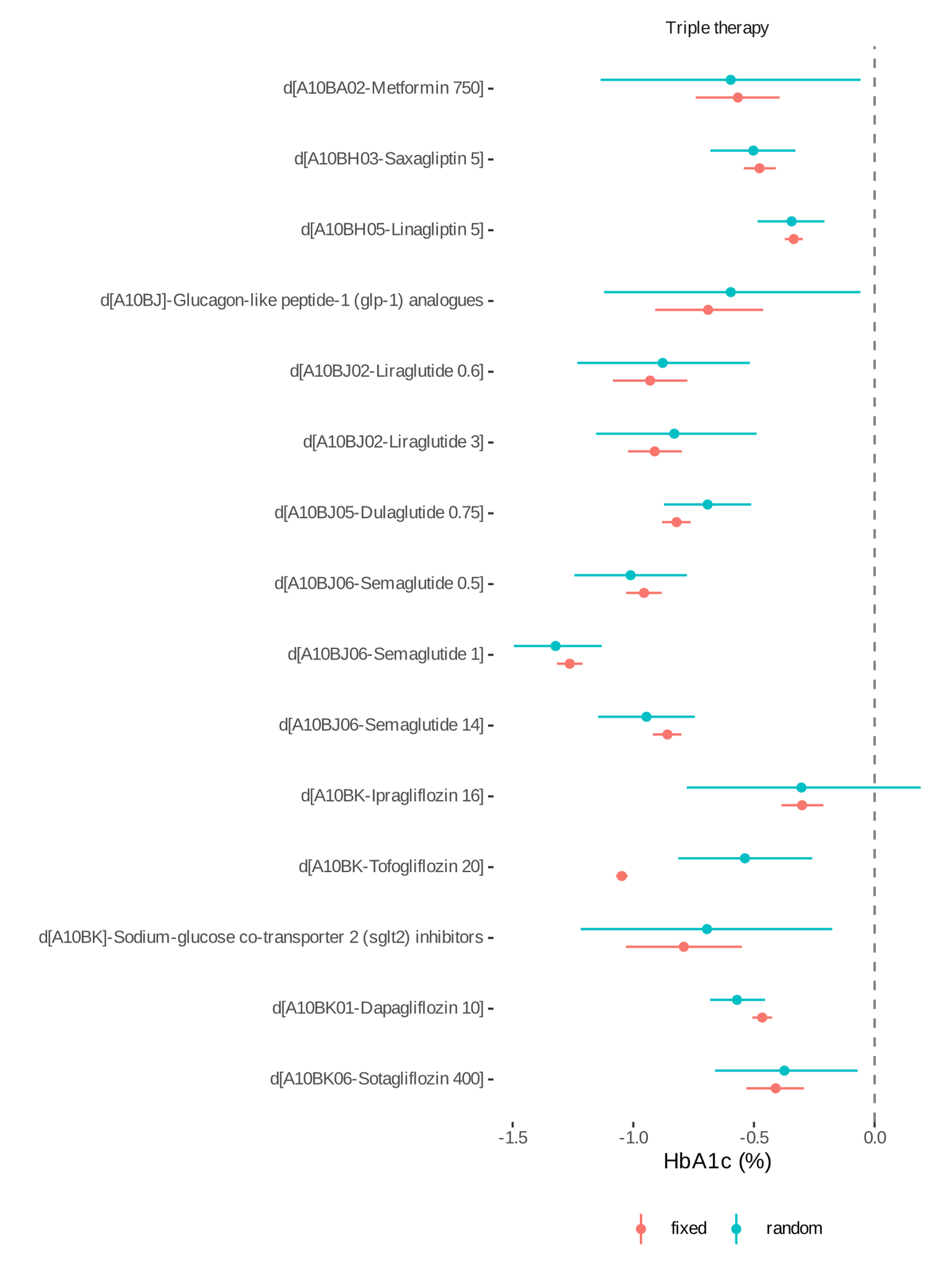


###### Main treatment effects for each treatment versus placebo based on a standard network meta-analysis without covariates. The point estimates are the means and line-ranges indicate the 95% credible intervals. The plots are separated into mono-, dual- and triple-therapy. Red ink indicates fixed effects models and blue ink random effects models. The majority of treatments reduced HbA1c; the difference ranged from -0.5% to -1.5%. Random sample of treatments shown here. Full plots available at <https://github.com/Type2DiabetesSystematicReview/nma_agesex_public/tree/main/Outputs/ef1.pdf>

### eFigure 3 Main treatment effects for MACE outcome by class


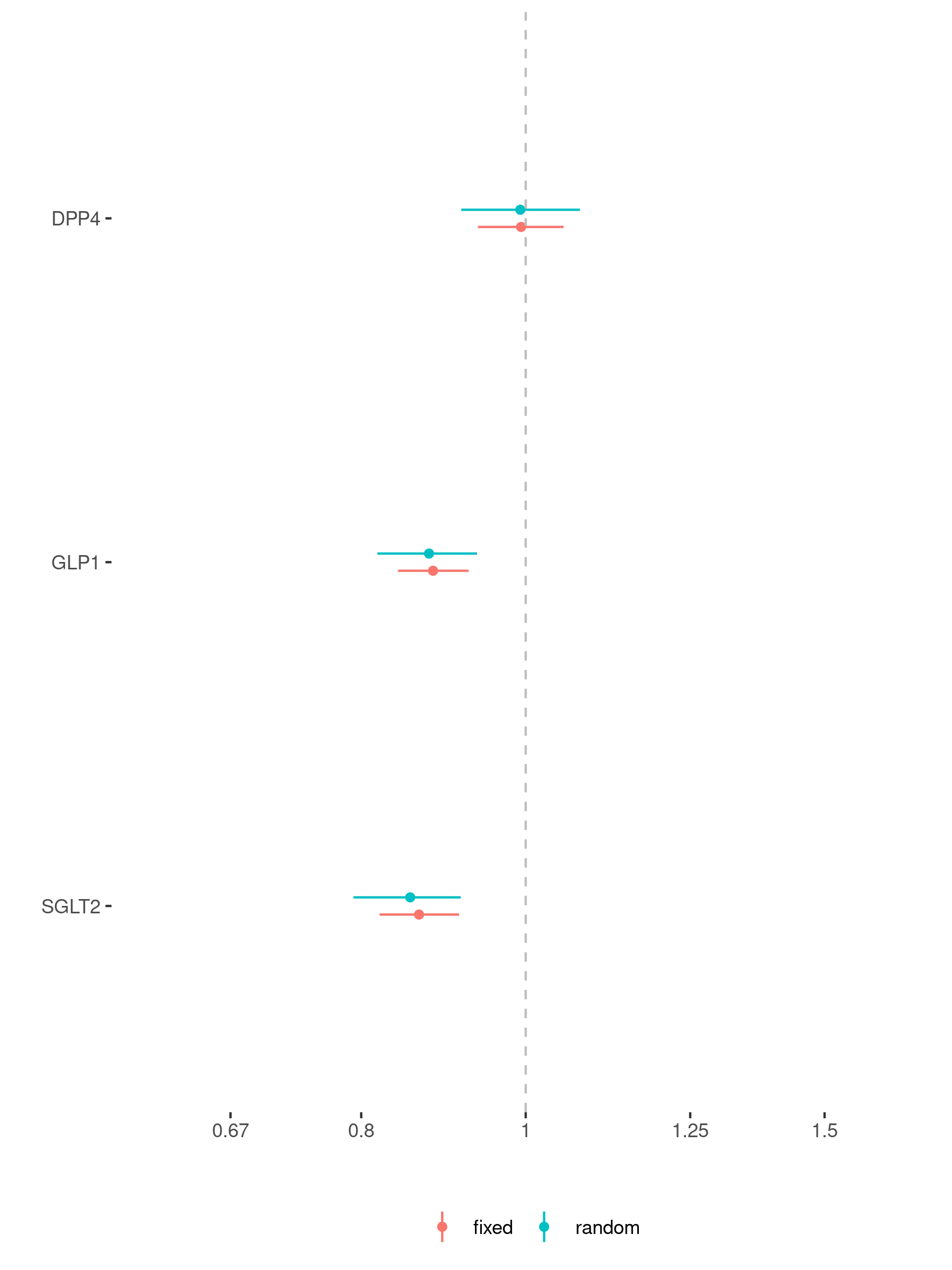


As eFigure 2, but for the MACE outcome and effect estimates as hazard ratios.

### eFigure 4 Non-linear age-treatment interaction estimates for each class for HbA1c


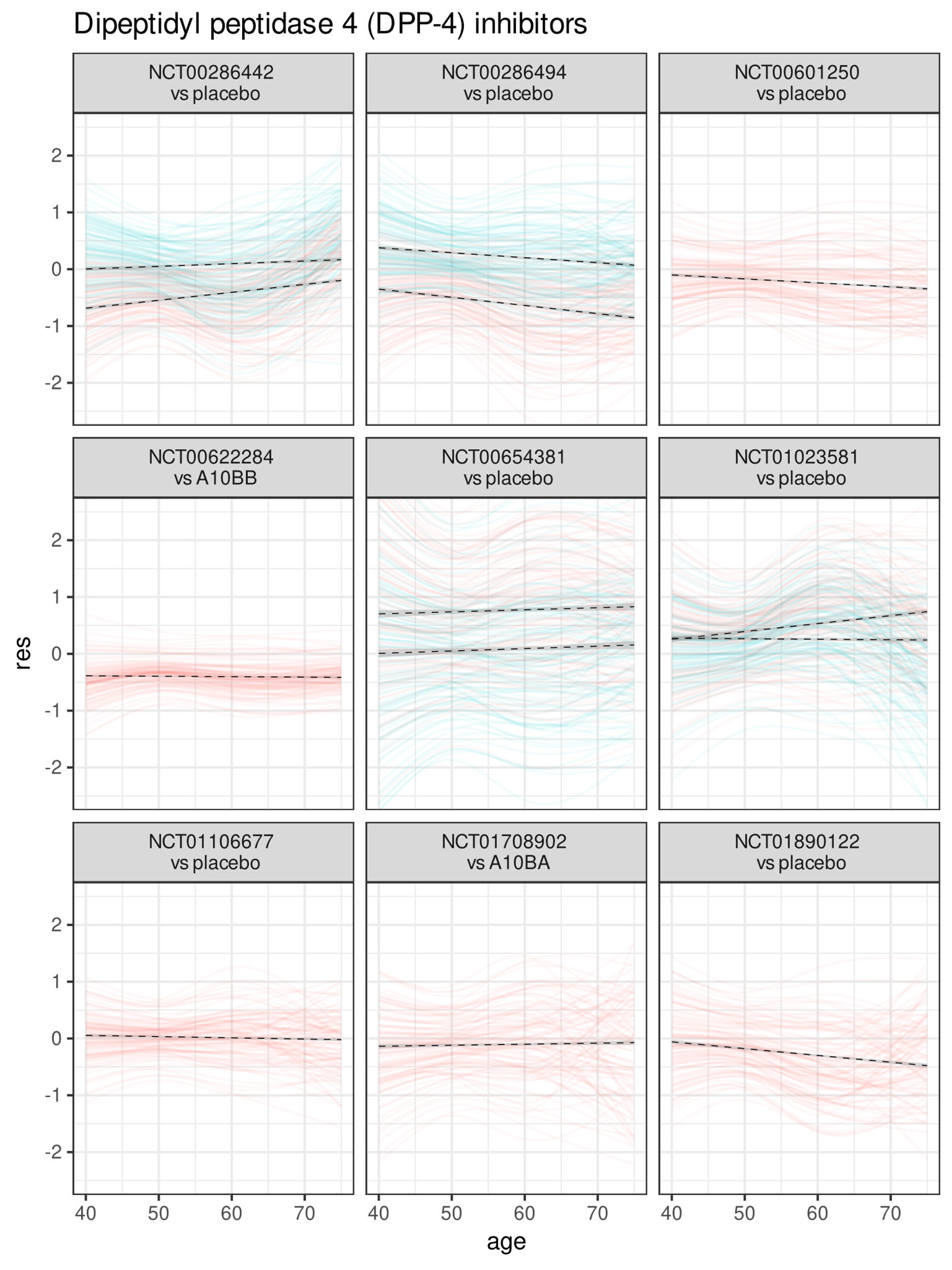


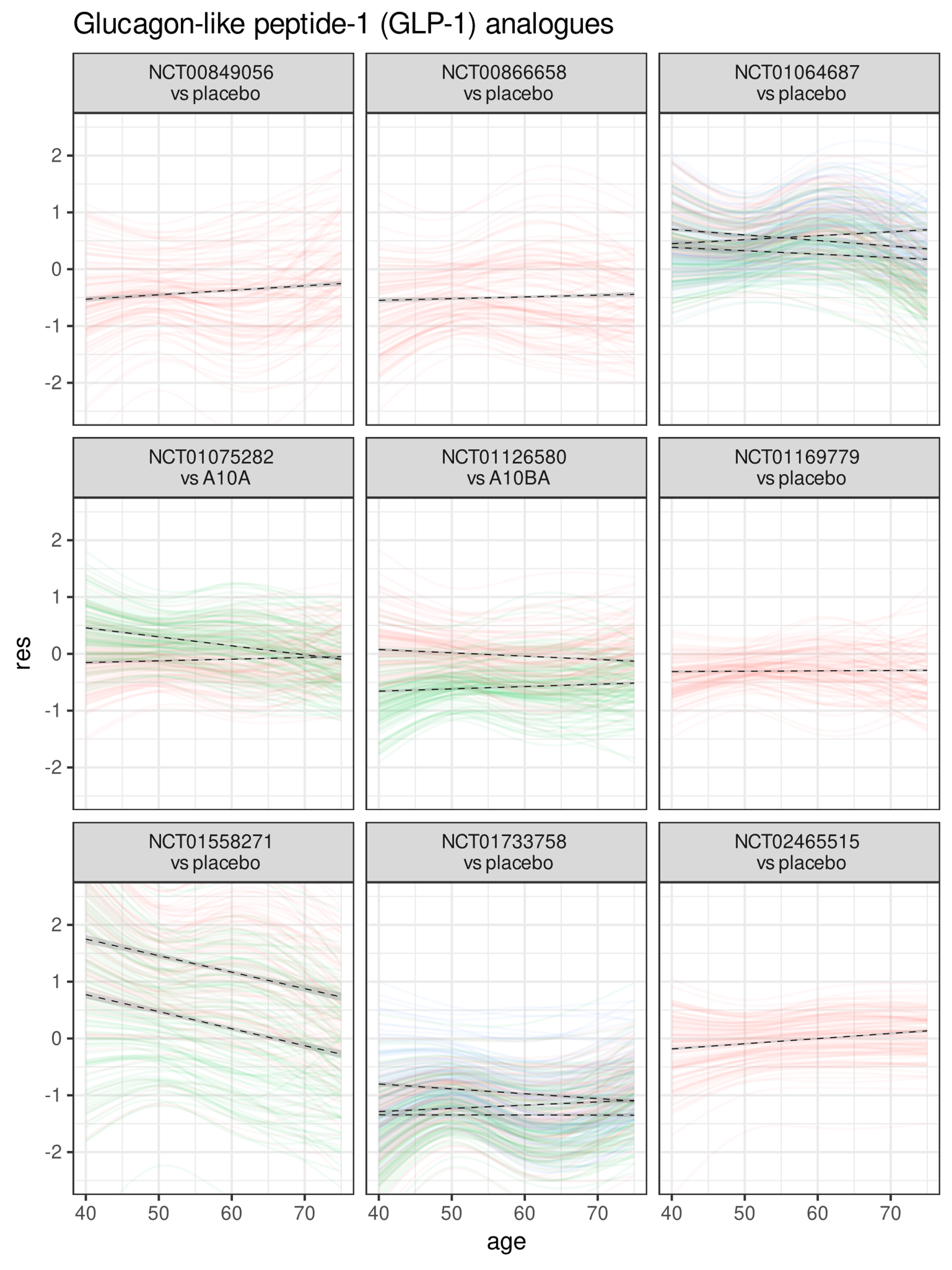


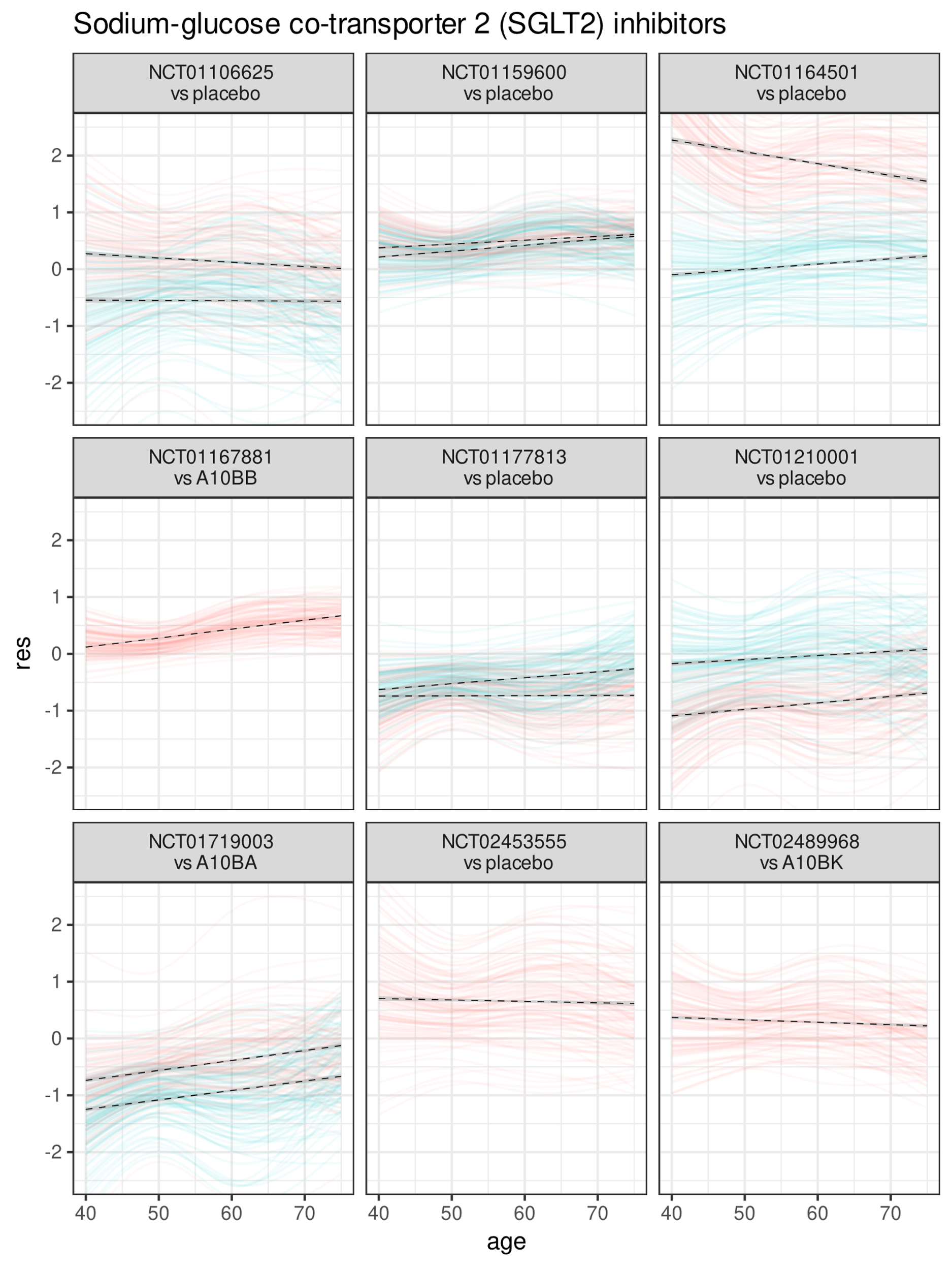


###### Plots of associations between age and variation in treatment efficacy using restricted cubic splines in order to examine for non-linearity for IPD HbA1c trials. Random sample of trial shown here. Full plots available at <https://github.com/Type2DiabetesSystematicReview/nma_agesex_public/tree/main/Outputs/ef4.pdf>

### eFigure 5 Various sensitivity analyses for age-treatment and sex-treatment interaction for HbA1c


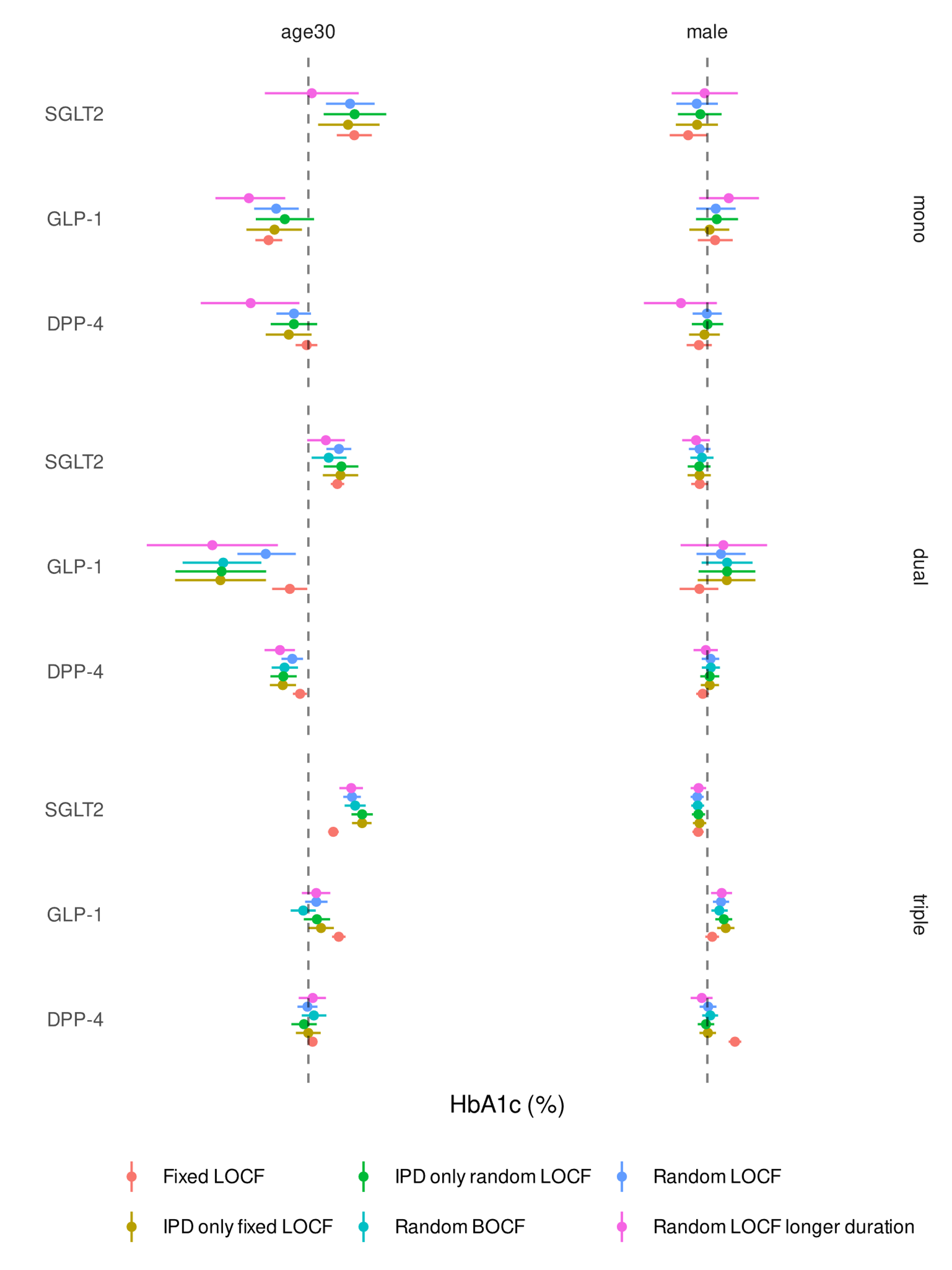


###### Plots of association between age and variation in treatment efficacy using restricted cubic splines in order to examine for non-linearity for IPD MACE trials. Fixed LOCF and random LOCF are shown in Figure 2 in the main manuscript and repeated her for the purposes of comparison. BOCF indicates baseline observation carried forward and LOCF last observation carried forward. Longer duration indicates trials with >= 26 weeks follow-up

### eFigure 6 Various sensitivity analyses for age-treatment and sex-treatment interaction for MACE


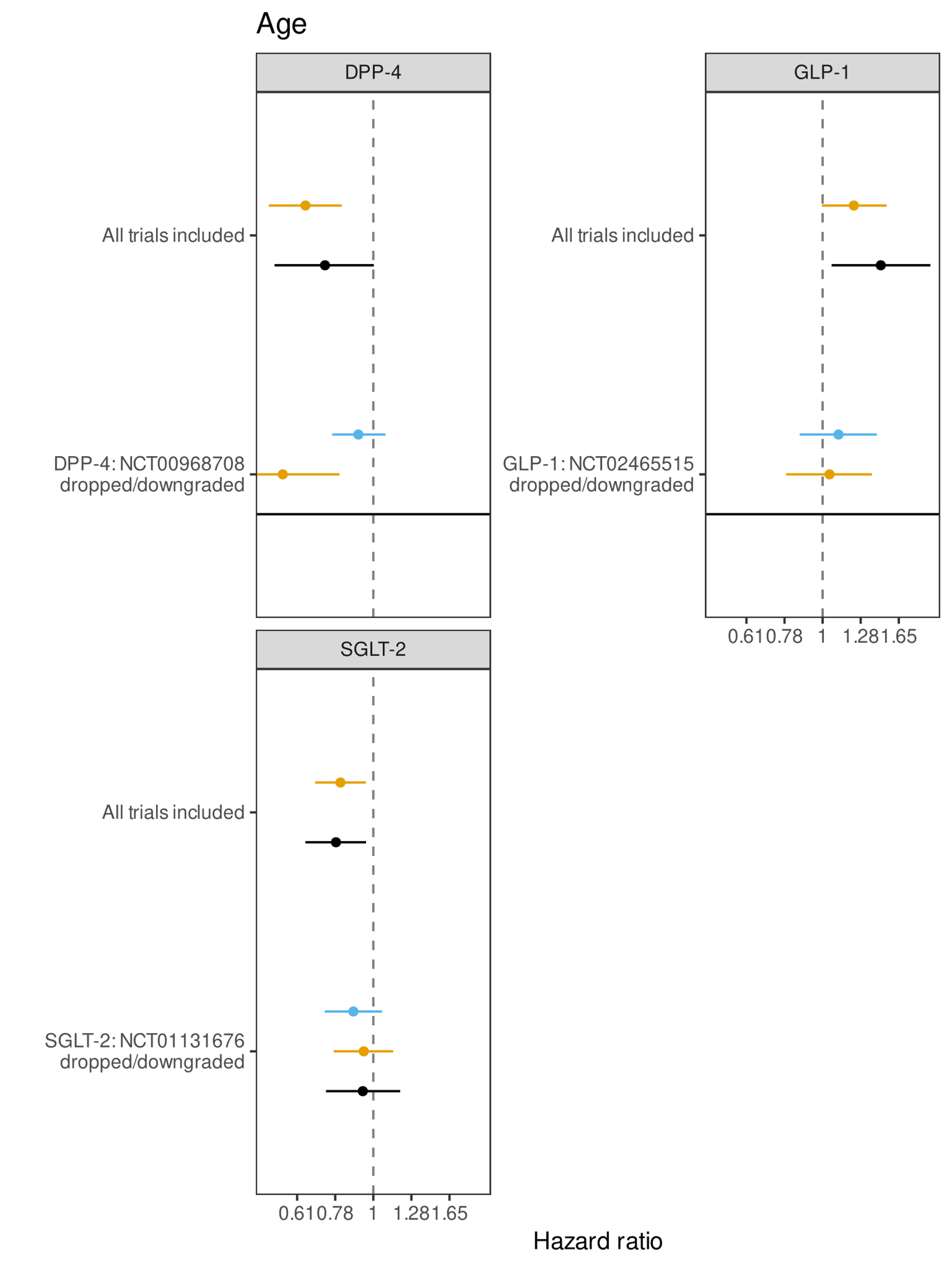


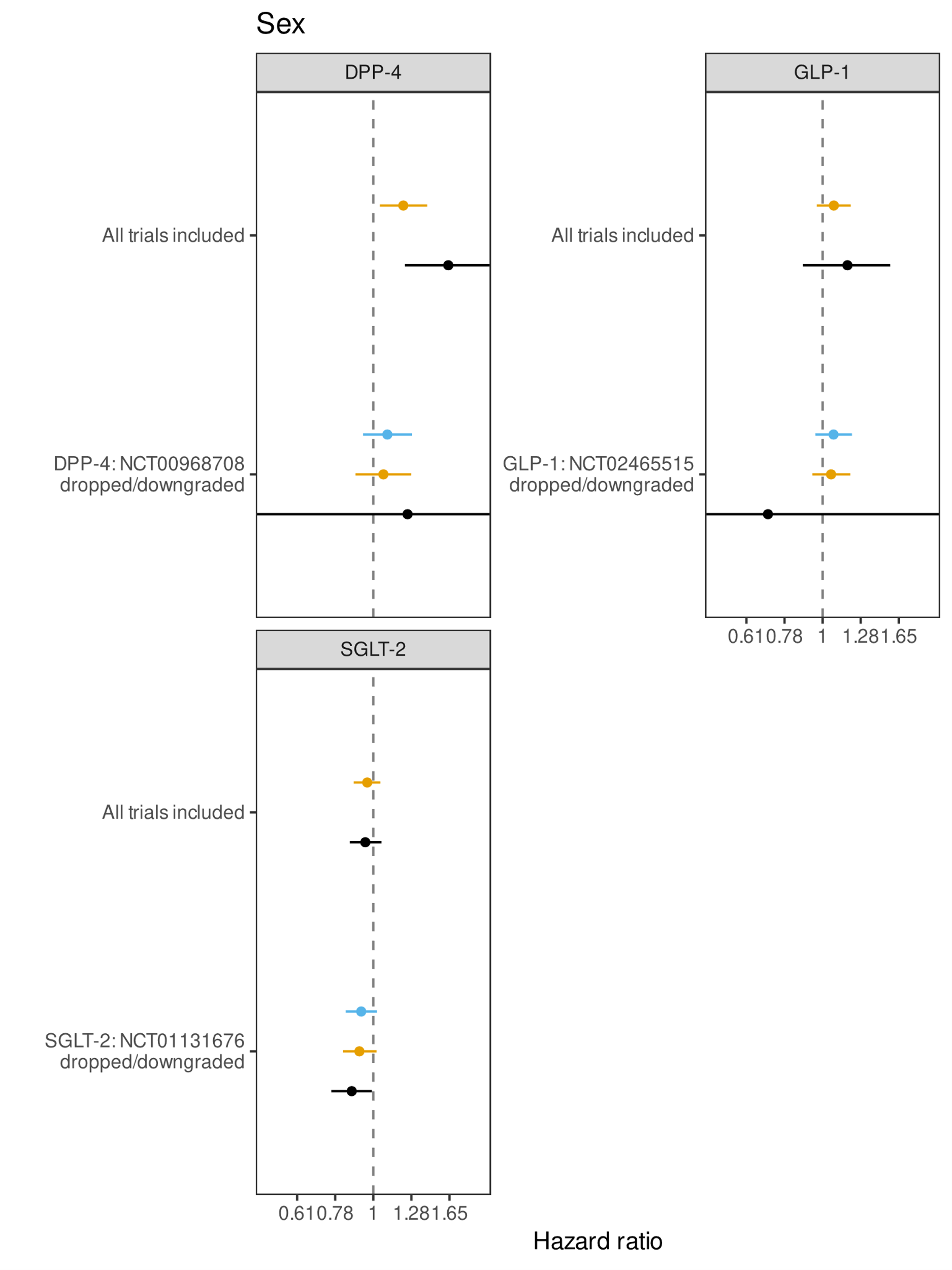


###### As Figure 2a in the main manuscript, but with various sensitivity analyses as described in the eMethods. Black ink indicates no data for dropped/downgraded trial AND no subgroup data for any trial. Gold ink indicates no data for dropped/downgraded trial AND subgroup data included where available for other trials. Blue ink indicates subgroup data rather than IPD for dropped/downgraded trial AND subgroup data included where available for other trials.

### eFigure 7 Non-linear age-treatment interaction estimates for MACE trials


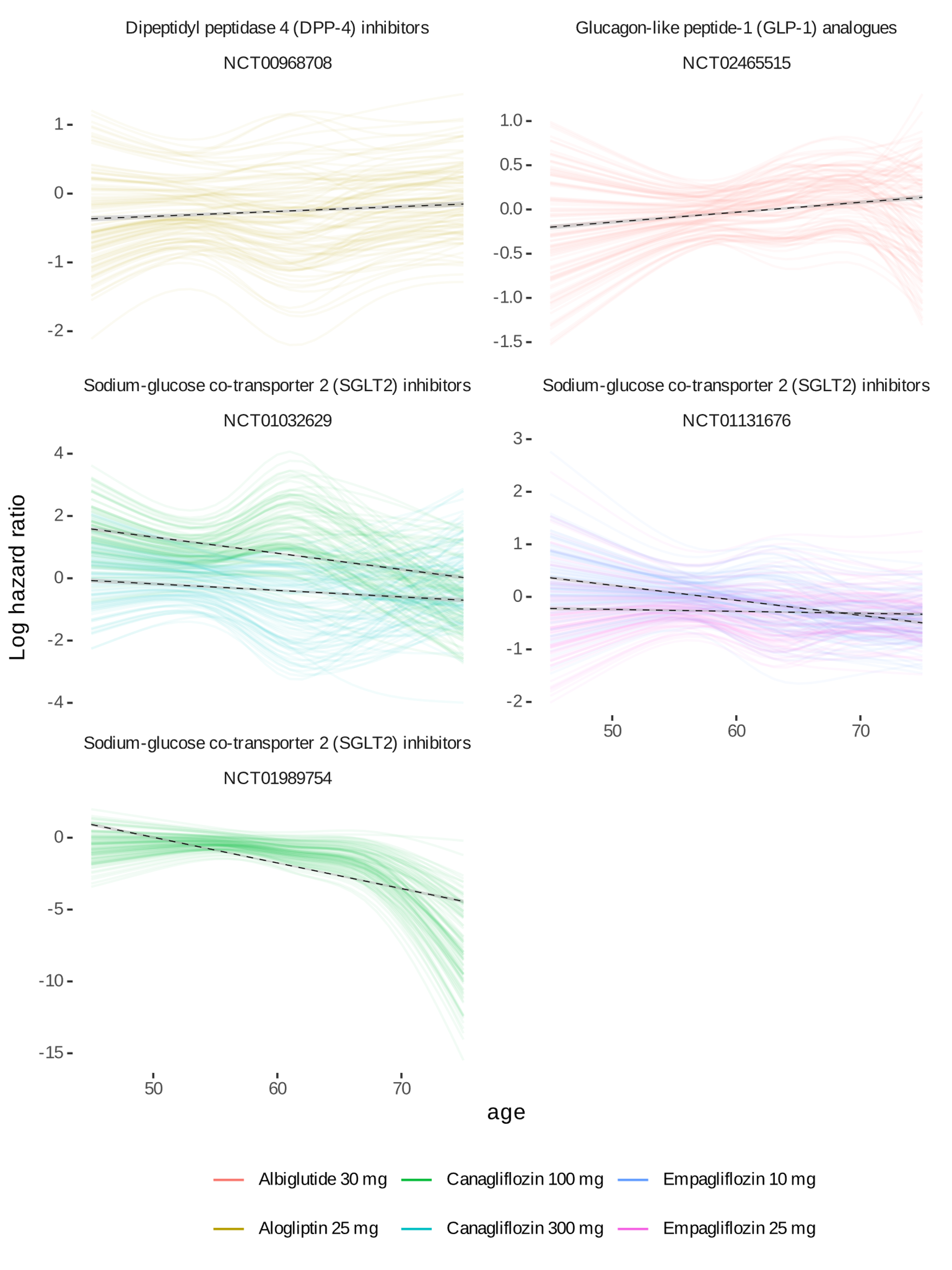


###### Plots of associations between age and variation in treatment efficacy for MACE using restricted cubic splines in order to examine for non-linearity for IPD.

### eFigure 8 Posterior distribution for age-treatment interaction for SGLT-2 inhibitor versus placebo for HbA1c and MACE. Main analysis includes all trials while sensitivity analysis includes only those trials with both HBA1c and MACE results


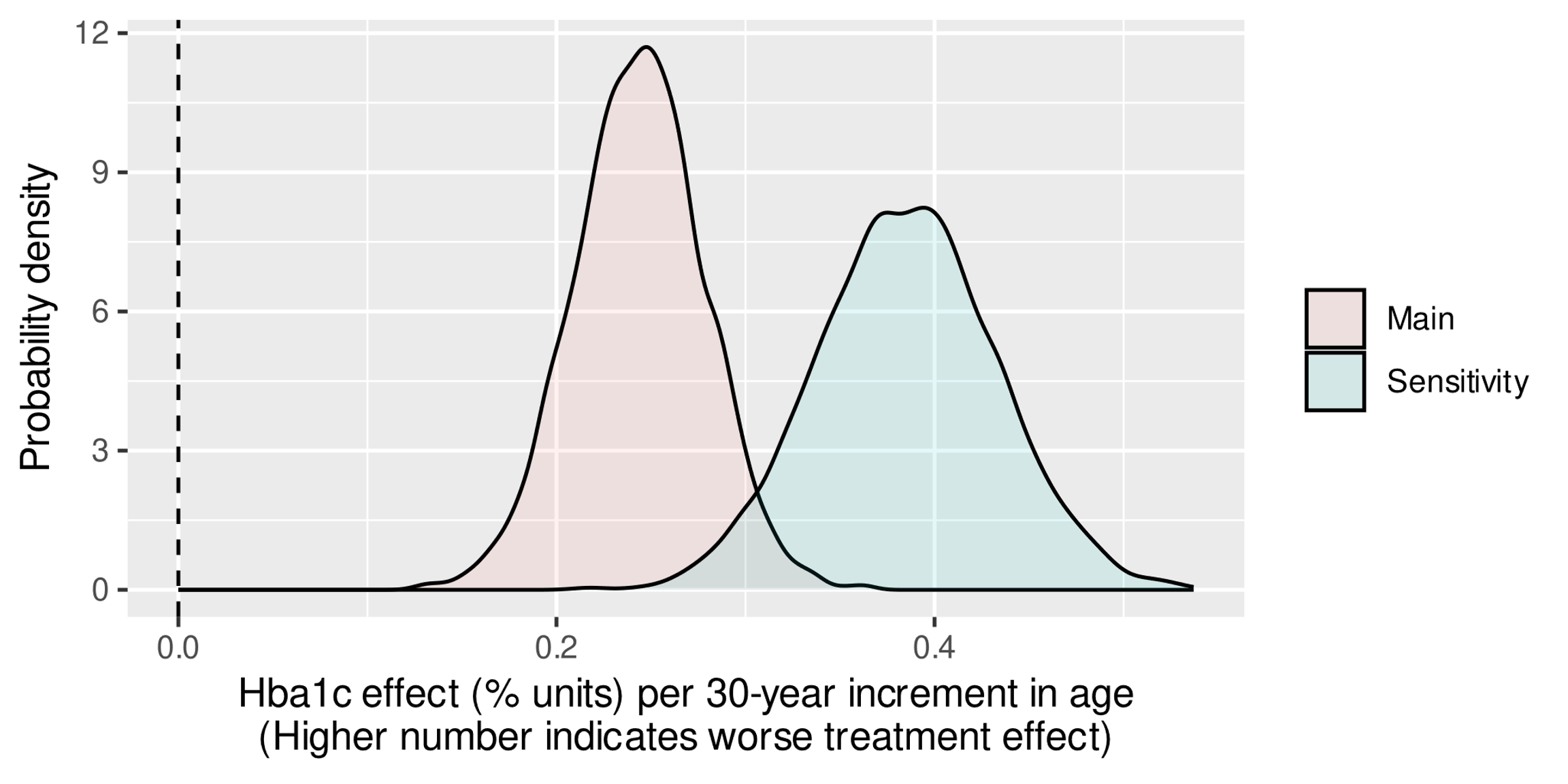


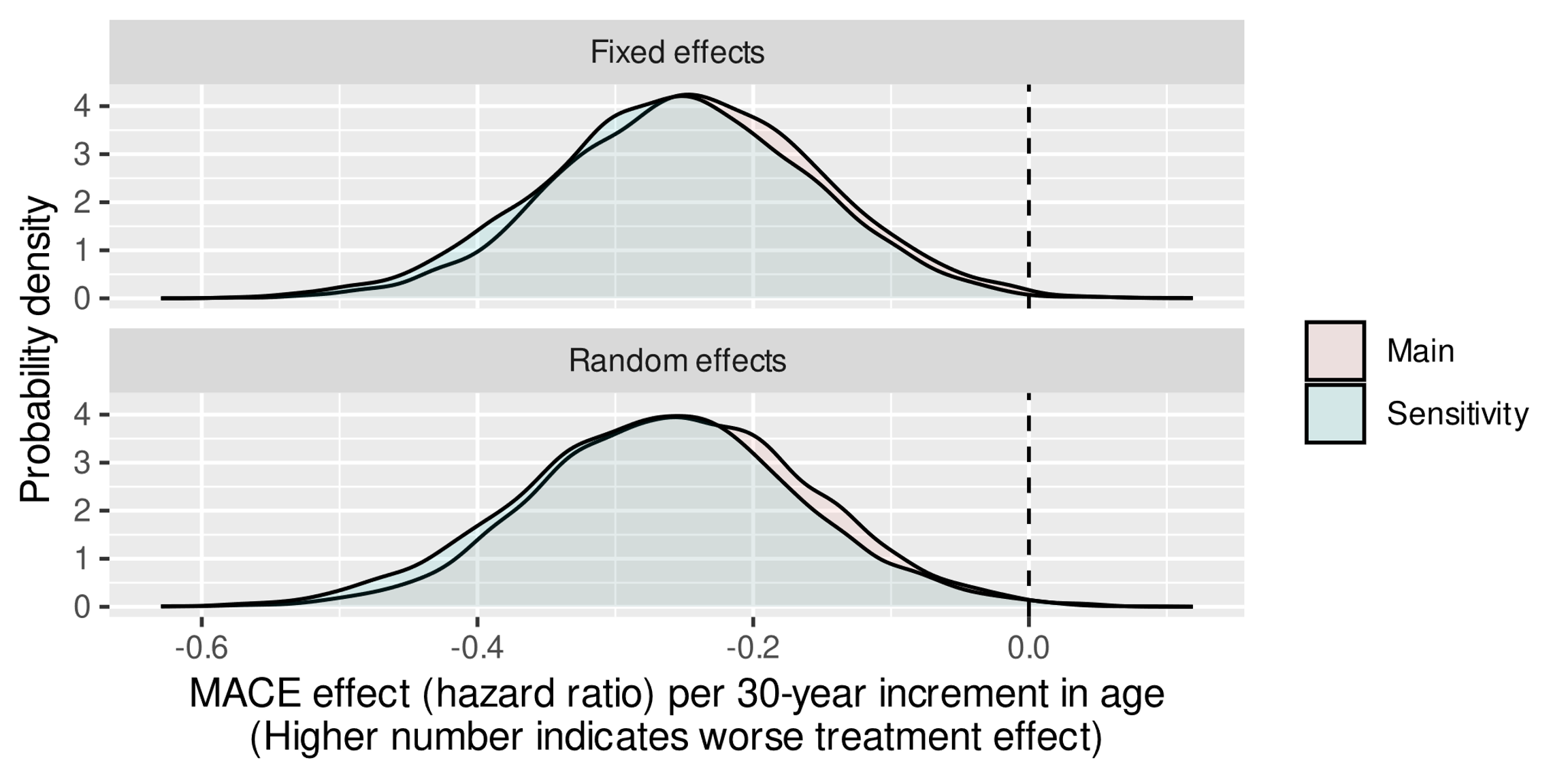


###### The posterior distributions for age-treatment interaction for each SGLT-2 inhibitors versus placebo for both outcomes, for both the main analysis and this sensitivity analysis. The distribution was somewhat broader for HbA1c, reflecting the smaller number of trials, but also showed lower efficacy with age. For MACE the results were very similar.

### eFigure 9a Age-treatment interactions (per 30-years) for adverse events


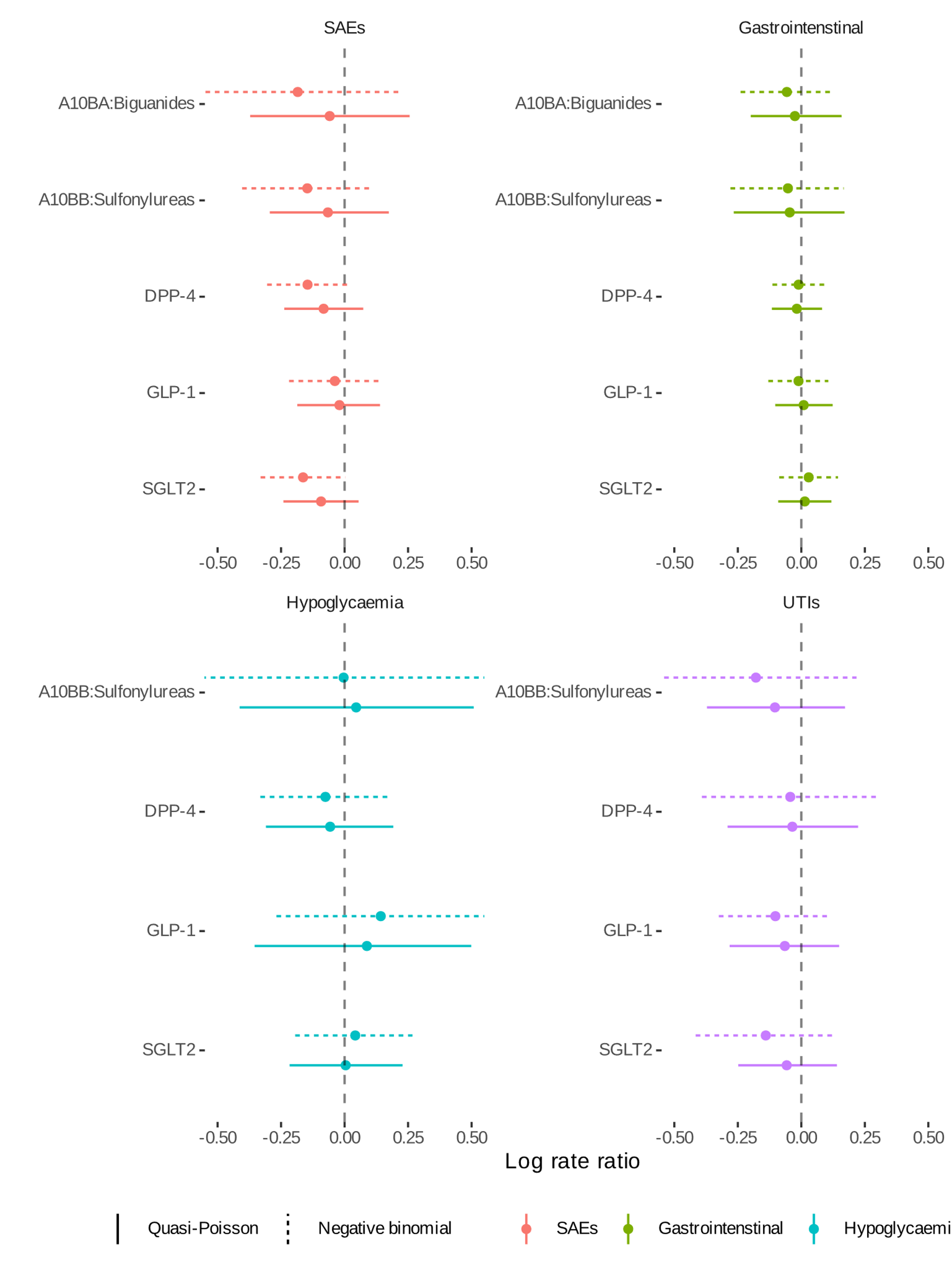


###### Dashed lines indicate that trial-level models were fit using negative binomial regression and solid lines indicate trial-level models were fit using quasi-Poisson regression.

### eFigure 9b Sex-treatment interactions for adverse events


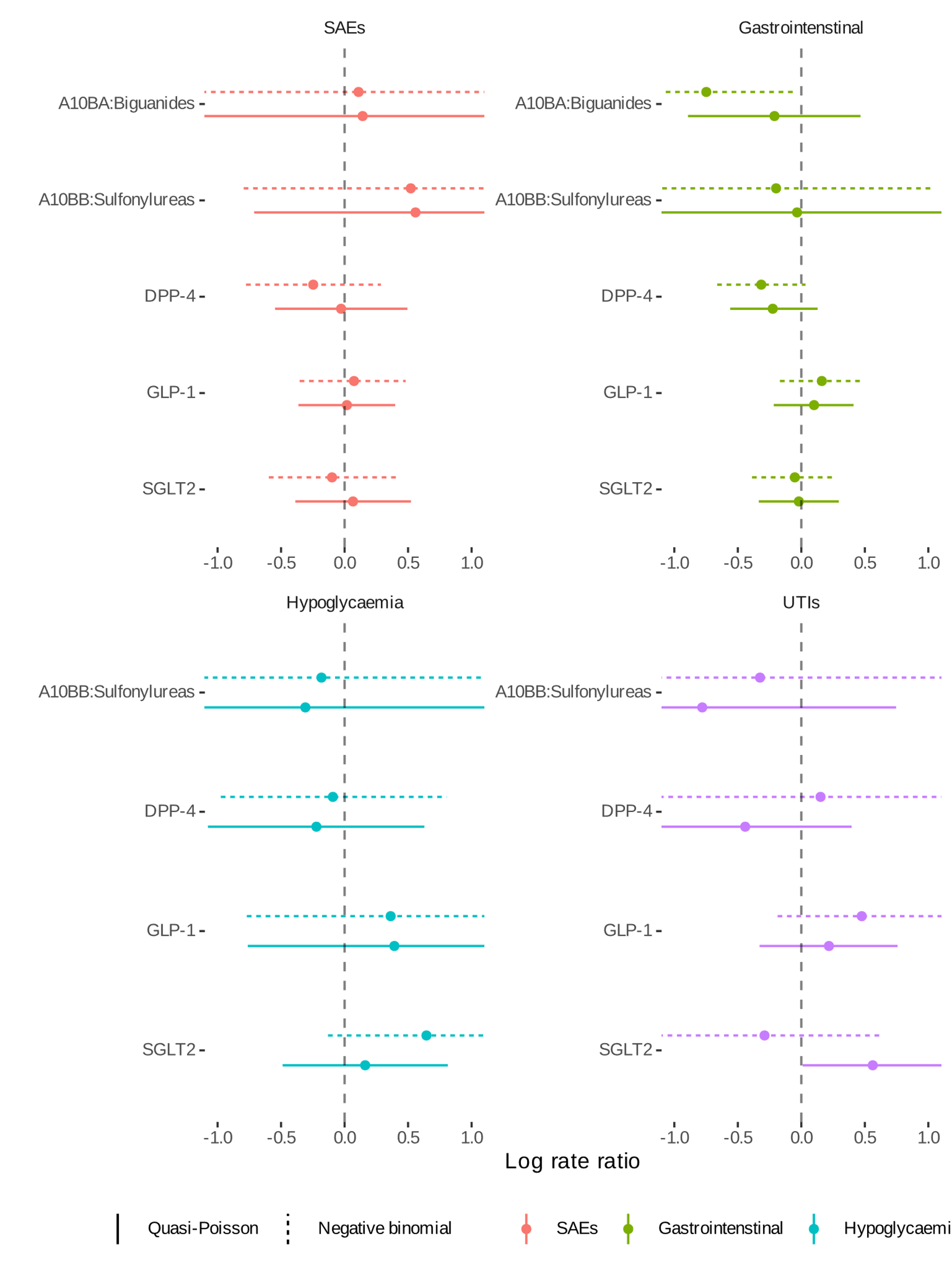


###### Dashed lines indicate that trial-level models were fit using negative binomial regression and solid lines indicate trial-level models were fit using quasi-Poisson regression.

### eFigure 10 Histogram of mortality for IPD trials
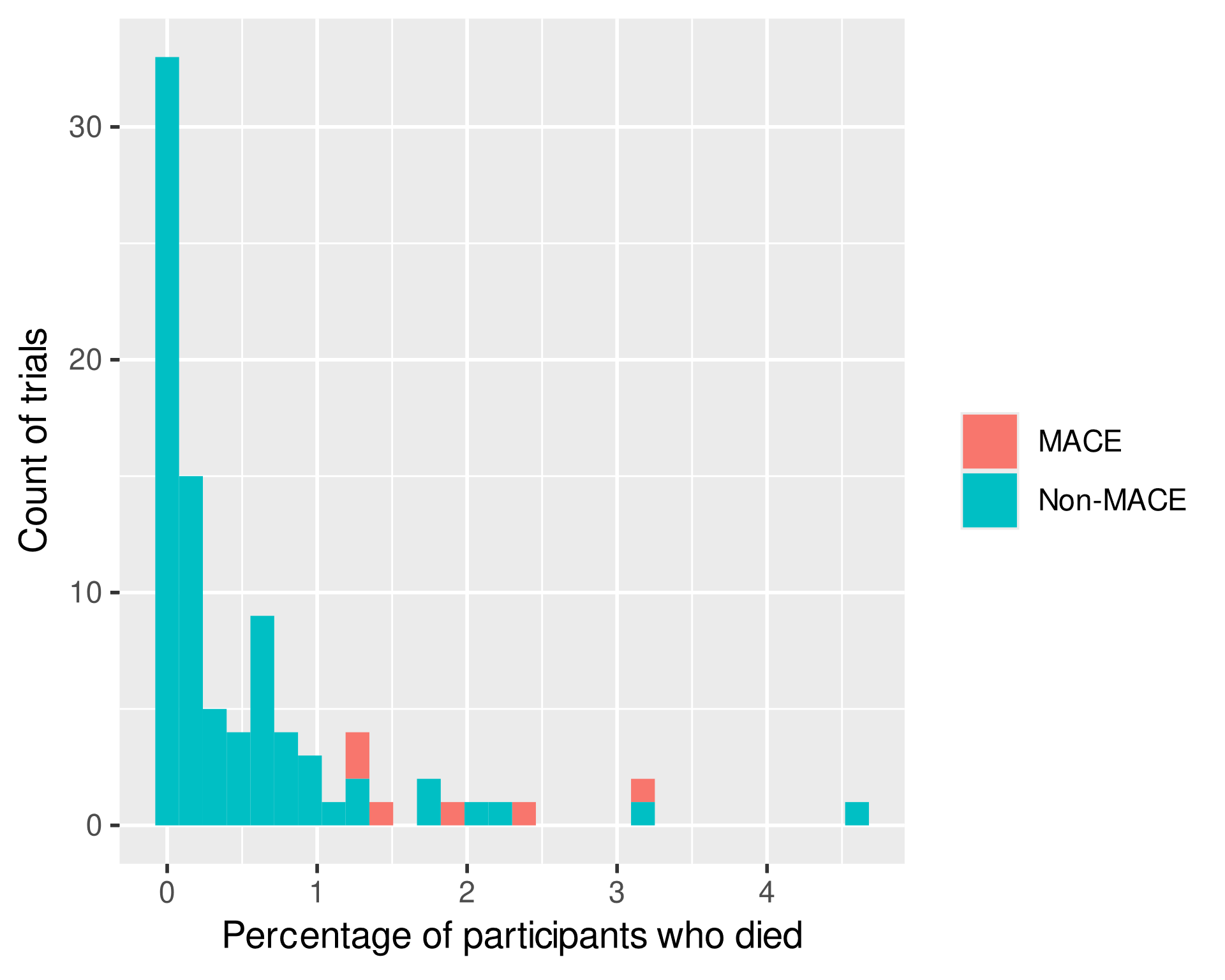


###### Shows the distribution of deaths (occurring prior to MACE if MACE occurred) across trials.

### eFigure 11 Hazard ratios for non-cardiovascular death in MACE IPD trials


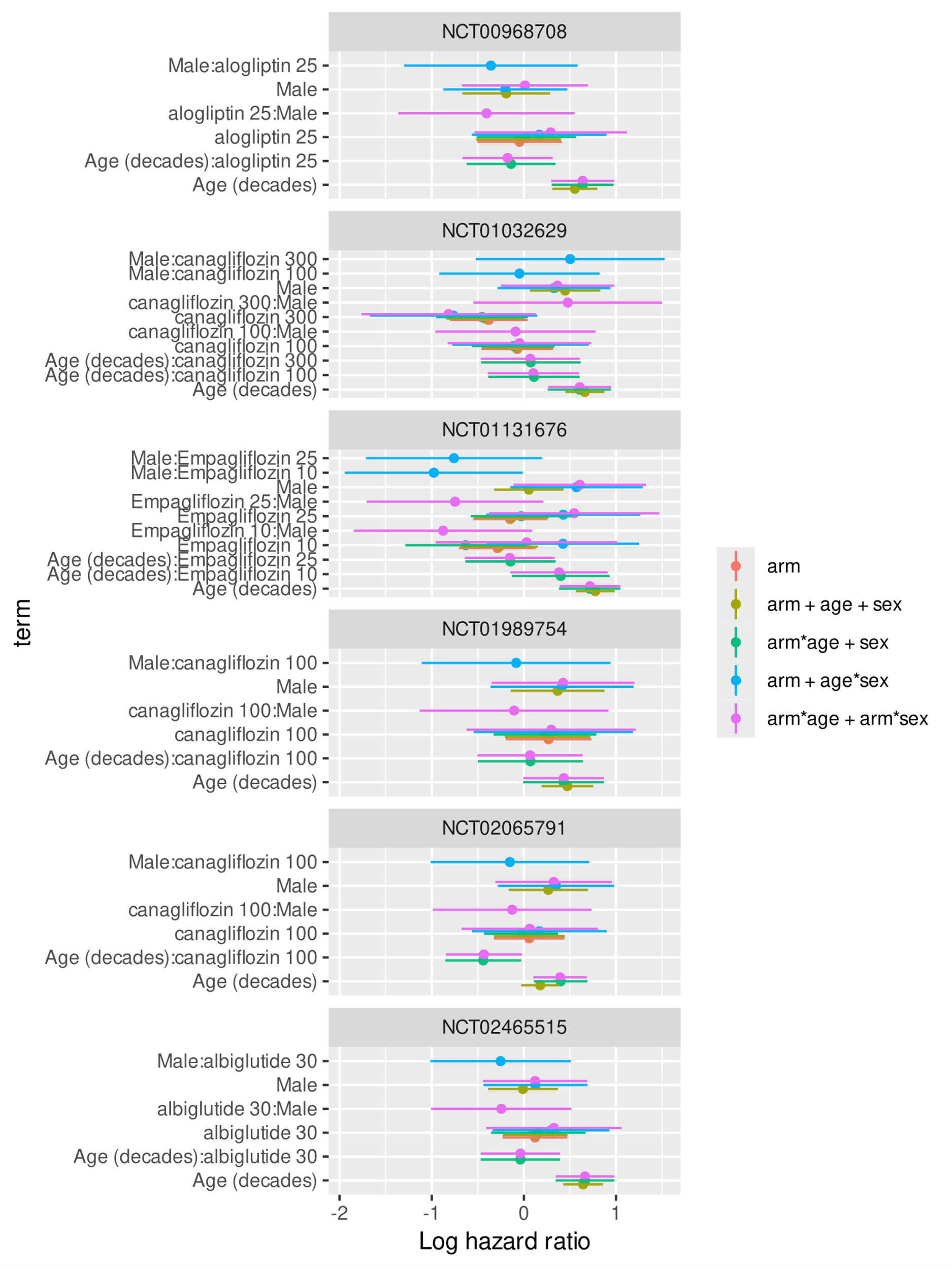


###### Estimated associations between, age, sex, treatment and treatment-covariate interactions and death (occurring prior to MACE if MACE occurred) for the MACE trials.

### eFigure 12 Main treatment effects for MACE


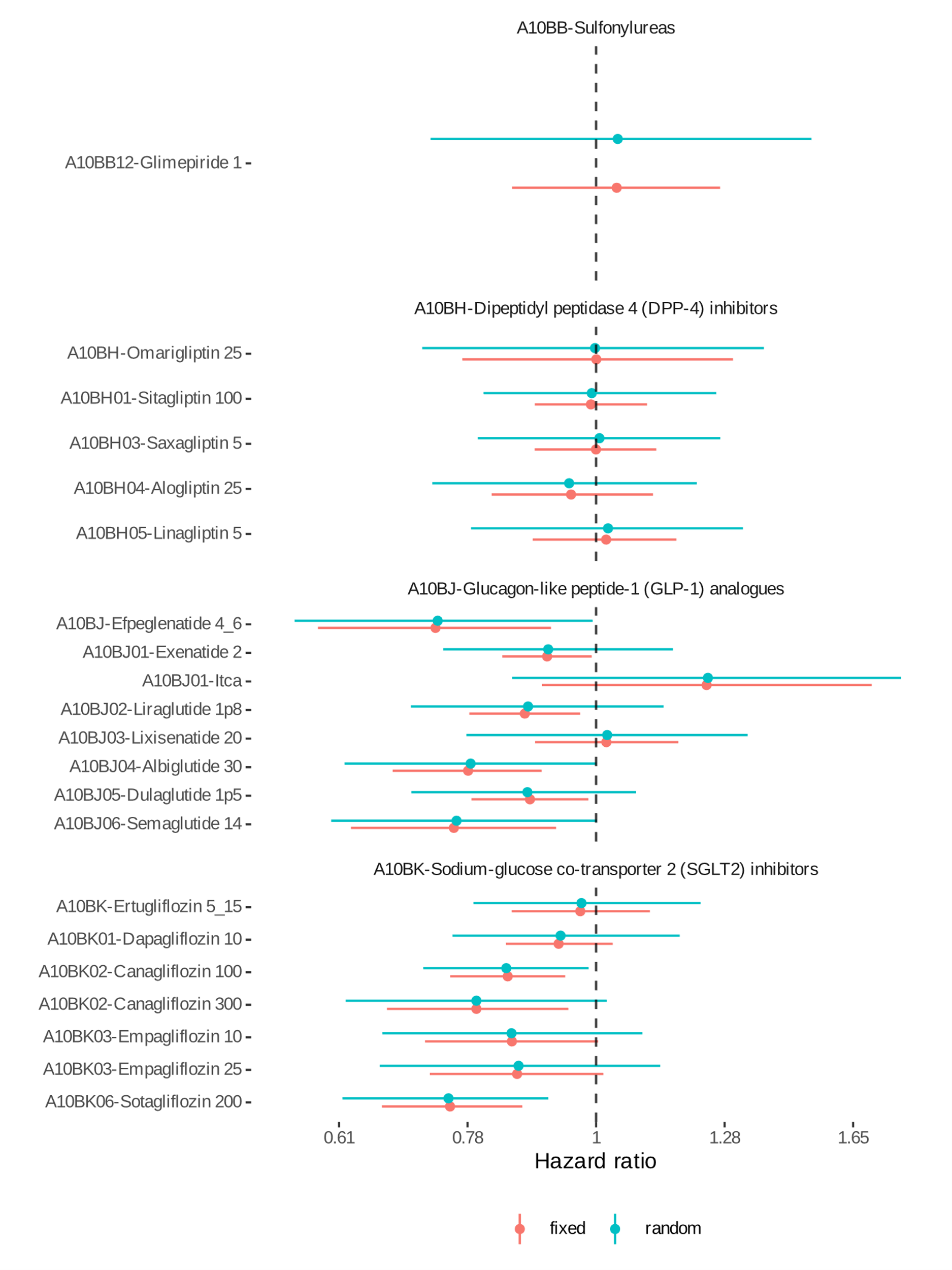


As eFigure 3, for individual treatments rather than drug classes.

# eTables

### eTable 1 Mean age in years by sex and arm for IPD MACE trials

| nct_id | arm | F | M | Difference |
| --- | --- | --- | --- | --- |
| NCT00968708 | alogliptin | 65.7 | 63.6 | 2.1 |
| NCT00968708 | placebo | 65.0 | 62.3 | 2.7 |
| NCT01032629 | JNJ-28431754-100 mg | 63.3 | 61.8 | 1.5 |
| NCT01032629 | JNJ-28431754-300 mg | 63.9 | 62.5 | 1.4 |
| NCT01032629 | PLACEBO | 64.5 | 62.6 | 1.9 |
| NCT01131676 | BI 10773 10mg | 64.1 | 63.5 | 0.6 |
| NCT01131676 | BI 10773 25mg | 65.8 | 64.5 | 1.2 |
| NCT01131676 | Placebo | 67.5 | 65.1 | 2.4 |
| NCT01989754 | Canagliflozin | 66.6 | 63.6 | 3.0 |
| NCT01989754 | Placebo | 66.8 | 65.1 | 1.7 |
| NCT02465515 | ALBIGLUTIDE | 67.0 | 65.6 | 1.4 |
| NCT02465515 | PLACEBO | 66.9 | 64.8 | 2.1 |

###

### eTable 2 Between-study heterogeneity standard deviation “tau” for main effects

|  |  |  |  | Quantiles of posterior distribution | | | | |  |  |
| --- | --- | --- | --- | --- | --- | --- | --- | --- | --- | --- |
| Model name | Est | SE | SD | 2.5% | 25% | 50% | 75% | 97.5 | Effective sample size | Rhat |
| m02_aggipd_random_dual_f4_ge12 | 0.198 | 0.001 | 0.015 | 0.170 | 0.187 | 0.197 | 0.207 | 0.228 | 563.223 | 1.007 |
| m03_aggipd_random_dual_f8_ge12 | 0.855 | 0.002 | 0.045 | 0.769 | 0.824 | 0.853 | 0.884 | 0.948 | 407.960 | 1.005 |
| m04_aggipd_random_dual_f4_ge26 | 0.154 | 0.001 | 0.024 | 0.111 | 0.137 | 0.152 | 0.169 | 0.205 | 898.266 | 1.000 |
| m06_aggipd_random_mono_f4_ge12 | 0.226 | 0.001 | 0.028 | 0.178 | 0.207 | 0.225 | 0.244 | 0.284 | 1371.153 | 1.001 |
| m08_aggipd_random_mono_f4_ge26 | 0.213 | 0.003 | 0.072 | 0.104 | 0.163 | 0.203 | 0.252 | 0.391 | 554.015 | 1.004 |
| m10_aggipd_random_triple_f4_ge12 | 0.246 | 0.000 | 0.014 | 0.219 | 0.236 | 0.245 | 0.255 | 0.276 | 1091.021 | 1.002 |
| m11_aggipd_random_triple_f8_ge12 | 1.012 | 0.002 | 0.045 | 0.924 | 0.981 | 1.011 | 1.042 | 1.104 | 519.355 | 1.001 |
| m12_aggipd_random_triple_f4_ge26 | 0.212 | 0.000 | 0.018 | 0.179 | 0.200 | 0.211 | 0.223 | 0.248 | 1622.576 | 1.002 |
| m14_ipd_random_dual_f4_ge12 | 0.069 | 0.001 | 0.023 | 0.026 | 0.054 | 0.068 | 0.083 | 0.118 | 1358.449 | 1.001 |
| m16_ipd_random_mono_f4_ge12 | 0.403 | 0.005 | 0.132 | 0.227 | 0.314 | 0.378 | 0.464 | 0.721 | 810.213 | 1.002 |
| m18_ipd_random_triple_f4_ge12 | 0.153 | 0.000 | 0.019 | 0.119 | 0.140 | 0.152 | 0.165 | 0.194 | 1589.518 | 1.001 |
| m19_aggipd_random_dual_f1_ge12 | 0.194 | 0.000 | 0.014 | 0.168 | 0.184 | 0.193 | 0.203 | 0.222 | 1449.817 | 1.002 |
| m20_aggipd_random_mono_f1_ge12 | 0.238 | 0.001 | 0.027 | 0.191 | 0.219 | 0.236 | 0.255 | 0.299 | 1405.105 | 1.000 |
| m21_aggipd_random_triple_f1_ge12 | 0.239 | 0.000 | 0.014 | 0.214 | 0.229 | 0.239 | 0.248 | 0.267 | 1495.502 | 1.003 |
| random_mace_agesex_age | 0.139 | 0.002 | 0.061 | 0.027 | 0.098 | 0.135 | 0.173 | 0.271 | 602.568 | 1.003 |
| random_mace_agesex_main | 0.302 | 0.011 | 0.195 | 0.064 | 0.174 | 0.255 | 0.373 | 0.800 | 309.755 | 1.020 |
| random_mace_agesex_noipd | 1.340 | 0.049 | 1.056 | 0.052 | 0.505 | 1.114 | 1.925 | 3.963 | 459.261 | 1.009 |
| random_mace_agesex_sex | 0.118 | 0.004 | 0.072 | 0.007 | 0.064 | 0.112 | 0.159 | 0.284 | 279.832 | 1.004 |
| random_mace_nointer | 0.078 | 0.004 | 0.064 | 0.003 | 0.028 | 0.061 | 0.114 | 0.229 | 244.882 | 1.012 |

###

### eTable 3 Adverse incident event counts and rates for IPD trials

| Trial ID | Amputation | DKA | Gastrointestinal | Hypo glycaemia | Serious adverse events | Urinary tract infection |
| --- | --- | --- | --- | --- | --- | --- |
| NCT00286429 | 0 | 0 | 65/51829 (45.81) [16.7%] | <10/58195 (1.26) [0.5%] | 21/56869 (13.49) [5.4%] | <10/57972 (1.89) [0.8%] |
| NCT00286442 | 0 | 0 | 61/80707 (27.61) [11.6%] | 0 | 19/86144 (8.06) [3.6%] | <10/86584 (1.69) [0.8%] |
| NCT00286455 | 0 | 0 | 41/49357 (30.34) [12.5%] | 0 | <10/52415 (5.57) [2.4%] | <10/52765 (2.77) [1.2%] |
| NCT00286468 | 0 | 0 | 70/74538 (34.3) [14%] | <10/82002 (0.89) [0.4%] | 23/80470 (10.44) [4.6%] | <10/81953 (0.89) [0.4%] |
| NCT00306384 | 0 | 0 | 794/2348300 (12.35) [28.4%] | <10/2949663 (0.02) [0.1%] | 461/2726778 (6.18) [16.5%] | 81/2900365 (1.02) [2.9%] |
| NCT00601250 | 0 | 0 | 73/110075 (24.22) [10.4%] | <10/116557 (2.51) [1.1%] | 26/115095 (8.25) [3.7%] | <10/116831 (1.25) [0.6%] |
| NCT00602472 | 0 | <10/174782 (0.42) [0.2%] | 149/160020 (34.01) [14.1%] | 218/149057 (53.42) [20.6%] | 46/171473 (9.8) [4.3%] | <10/174521 (1.26) [0.6%] |
| NCT00621140 | 0 | 0 | 25/81880 (11.15) [5%] | <10/84020 (0.43) [0.2%] | 17/83091 (7.47) [3.4%] | <10/83773 (1.74) [0.8%] |
| NCT00622284 | 0 | 0 | 320/814181 (14.36) [20.6%] | 277/826317 (12.24) [17.8%] | 210/893024 (8.59) [13.5%] | 30/962192 (1.14) [1.9%] |
| NCT00641043 | 0 | 0 | 33/60304 (19.99) [8.5%] | <10/63290 (1.73) [0.8%] | 12/62534 (7.01) [3.1%] | <10/63331 (0.58) [0.3%] |
| NCT00654381 | 0 | 0 | 111/156799 (25.86) [19.8%] | <10/190254 (0.19) [0.2%] | <10/189045 (1.55) [1.4%] | <10/189631 (0.77) [0.7%] |
| NCT00688701 | 0 | 0 | 109/23691 (0.46) [30.28%] | 0 | <10/29093 (0.02) [1.67%] | <10/28930 (0.02) [1.39%] |
| NCT00707031 | 0 | 0 | 357/167162 (0.21) [56.31%] | <10/307016 (0) [1.1%] | 43/298982 (0.01) [6.78%] | 31/299728 (0.01) [4.89%] |
| NCT00707993 | 0 | 0 | 79/110860 (26.03) [17.9%] | <10/127705 (2) [1.6%] | 30/123366 (8.88) [6.8%] | <10/127254 (2.01) [1.6%] |
| NCT00712673 | 0 | 0 | 335/215614 (0.16) [49.26%] | <10/362016 (0) [0.15%] | 47/349213 (0.01) [6.91%] | 37/347862 (0.01) [5.44%] |
| NCT00713830 | 0 | 0 | 372/291077 (0.13) [43.36%] | 36/433950 (0.01) [4.2%] | 77/428640 (0.02) [8.97%] | 33/437438 (0.01) [3.85%] |
| NCT00715624 | 0 | <10/244245 (0) [0.2%] | 218/157111 (0.14) [44.13%] | 28/236187 (0.01) [5.67%] | 46/232702 (0.02) [9.31%] | 28/233441 (0.01) [5.67%] |
| NCT00740051 | 0 | 0 | 39/65163 (0.06) [17.18%] | 0 | <10/72859 (0.01) [1.76%] | <10/70779 (0.01) [3.96%] |
| NCT00763451 | 0 | 0 | 239/153125 (0.16) [49.59%] | <10/256084 (0) [0.83%] | 53/246697 (0.02) [11%] | 33/246777 (0.01) [6.85%] |
| NCT00763815 | 0 | 0 | 216/161290 (0.13) [44.63%] | <10/258816 (0) [1.86%] | 31/251838 (0.01) [6.4%] | 40/246497 (0.02) [8.26%] |
| NCT00798161 | 0 | 0 | 109/115285 (34.53) [13.8%] | 11/126220 (3.18) [1.4%] | 45/125755 (13.07) [5.7%] | <10/126409 (1.73) [0.8%] |
| NCT00800683 | 0 | 0 | 43/30201 (0.14) [33.08%] | 0 | <10/38768 (0.02) [5.38%] | 14/38044 (0.04) [10.77%] |
| NCT00819091 | 0 | 0 | 14/28172 (0.05) [5.88%] | 0 | <10/29057 (0.01) [1.26%] | <10/28849 (0.02) [2.52%] |
| NCT00866658 | 0 | 0 | 119/34337 (0.35) [38.39%] | <10/48931 (0.01) [0.97%] | 19/48486 (0.04) [6.13%] | <10/48968 (0.01) [0.97%] |
| NCT00915772 | - | - | - | - | 77/184607 (15.23) [13.6%] | - |
| NCT00954447 | 0 | 0 | 177/480854 (13.44) [14.1%] | 288/434916 (24.19) [22.9%] | 83/518154 (5.85) [6.6%] | 15/536340 (1.02) [1.2%] |
| NCT00968708 | 0 | <10/2959107 (0.02) [0%] | 966/2550134 (13.84) [18%] | 332/2824073 (4.29) [6.2%] | 1831/2306424 (29) [34%] | 77/2932471 (0.96) [1.4%] |
| NCT00968812 | 0 | 0 | 362/426302 (0.08) [24.93%] | 0 | 91/500630 (0.02) [6.27%] | 113/487825 (0.02) [7.78%] |
| NCT00975286 | 0 | 0 | 133/55532 (0.24) [29.82%] | <10/70086 (0.01) [2.02%] | 25/69819 (0.04) [5.61%] | 14/69850 (0.02) [3.14%] |
| NCT00976937 | 0 | 0 | 90/40239 (0.22) [28.21%] | 0 | <10/51029 (0.01) [1.88%] | <10/50597 (0.02) [2.51%] |
| NCT00996658 | 0 | 0 | 26/41465 (0.06) [9.56%] | 0 | <10/43935 (0) [0.37%] | 13/42340 (0.03) [4.78%] |
| NCT01023581 | 0 | 0 | 105/120101 (31.93) [13.4%] | <10/133484 (0.27) [0.1%] | 16/132494 (4.41) [2%] | <10/132922 (1.92) [0.9%] |
| NCT01032629 | 0 | <10/6832130 (0) [0.16%] | 1756/4599516 (0.04) [40.55%] | <10/6830279 (0) [0.12%] | 1761/5330328 (0.03) [40.67%] | 635/6135470 (0.01) [14.67%] |
| NCT01064687 | 0 | 0 | 357/225081 (57.93) [36.5%] | <10/333777 (0.33) [0.3%] | 50/324491 (5.63) [5.1%] | <10/333036 (0.66) [0.6%] |
| NCT01075282 | <10/424051 (0.09) [0.1%] | 0 | 210/332983 (23.03) [25.3%] | <10/422948 (0.26) [0.4%] | 69/403079 (6.25) [8.3%] | 12/419321 (1.05) [1.4%] |
| NCT01081834 | 0 | 0 | 109/159386 (0.07) [18.57%] | 0 | 15/185145 (0.01) [2.56%] | 47/176262 (0.03) [8.01%] |
| NCT01084005 | 0 | 0 | 46/34092 (0.13) [19.09%] | 0 | <10/38598 (0.01) [1.66%] | 16/37598 (0.04) [6.64%] |
| NCT01087502 | 0 | 0 | 70/62517 (0.11) [29.79%] | <10/74838 (0.01) [1.7%] | <10/74735 (0.01) [2.55%] | 20/72039 (0.03) [8.51%] |
| NCT01106625 | 0 | 0 | 99/116712 (0.08) [21.11%] | 0 | 17/139344 (0.01) [3.62%] | 33/134617 (0.02) [7.04%] |
| NCT01106651 | 0 | 0 | 161/209036 (0.08) [22.49%] | 0 | 33/247788 (0.01) [4.61%] | 56/240216 (0.02) [7.82%] |
| NCT01106677 | 0 | 0 | 246/355369 (0.07) [19.16%] | 0 | 35/411500 (0.01) [2.73%] | 74/399273 (0.02) [5.76%] |
| NCT01106690 | 0 | 0 | 72/91508 (0.08) [20.93%] | 0 | 14/107281 (0.01) [4.07%] | 24/103408 (0.02) [6.98%] |
| NCT01126580 | 0 | 0 | 231/197119 (42.8) [28.6%] | 0 | 27/259771 (3.8) [3.3%] | <10/263358 (0.69) [0.6%] |
| NCT01137812 | 0 | <10/226635 (0) [0.13%] | 192/187780 (0.1) [25.4%] | <10/226381 (0) [0.13%] | 45/219343 (0.02) [5.95%] | 52/218163 (0.02) [6.88%] |
| NCT01159600 | 0 | 0 | 140/204552 (25) [10.6%] | 100/208176 (17.55) [7.6%] | 48/214140 (8.19) [3.6%] | 14/215663 (2.37) [1.1%] |
| NCT01164501 | 0 | <10/249875 (0.15) [0.1%] | 97/225492 (15.71) [13.1%] | 169/202924 (30.42) [22.8%] | 57/239679 (8.69) [7.7%] | 11/247552 (1.62) [1.5%] |
| NCT01167881 | 0 | 0 | 415/1397443 (10.85) [26.8%] | 231/1563094 (5.4) [14.9%] | 218/1620335 (4.91) [14.1%] | 59/1723127 (1.25) [3.8%] |
| NCT01169779 | 0 | 0 | 76/54019 (0.14) [19.49%] | 15/61639 (0.02) [3.85%] | <10/62753 (0.01) [1.79%] | <10/62769 (0.01) [1.03%] |
| NCT01177813 | 0 | 0 | 85/137409 (22.59) [9.5%] | <10/144395 (1.52) [0.7%] | 26/143273 (6.63) [2.9%] | <10/143963 (2.28) [1%] |
| NCT01191268 | 0 | 0 | 257/214360 (43.79) [29.1%] | 26/280701 (3.38) [2.9%] | 83/268194 (11.3) [9.4%] | <10/285120 (0.64) [0.6%] |
| NCT01194830 | 0 | 0 | 30/27987 (0.11) [13.89%] | 0 | <10/29756 (0.03) [4.17%] | 11/29704 (0.04) [5.09%] |
| NCT01204294 | 0 | 0 | 147/171656 (31.28) [25.6%] | 36/193454 (6.8) [6.3%] | 55/194672 (10.32) [9.6%] | 10/199210 (1.83) [1.7%] |
| NCT01210001 | 0 | 0 | 48/78336 (22.38) [9.6%] | 12/81012 (5.41) [2.4%] | 21/80978 (9.47) [4.2%] | 10/81449 (4.48) [2%] |
| NCT01214239 | 0 | 0 | 11/47399 (8.48) [3.7%] | <10/48436 (0.75) [0.3%] | <10/47862 (5.34) [2.3%] | 0 |
| NCT01215097 | 0 | 0 | 13/48679 (9.75) [4.2%] | <10/49790 (2.2) [1%] | <10/49720 (5.88) [2.6%] | 0 |
| NCT01289119 | 0 | <10/53858 (0.68) [0.2%] | 16/52953 (11.04) [3.2%] | 0 | <10/53684 (4.08) [1.2%] | 0 |
| NCT01306214 | 0 | <10/189840 (0.38) [0.4%] | 69/171334 (14.71) [12.2%] | 229/121750 (68.7) [40.5%] | 27/184583 (5.34) [4.8%] | <10/187745 (1.36) [1.2%] |
| NCT01368081 | 0 | 0 | 260/332028 (28.6) [23.7%] | 40/374892 (3.9) [3.6%] | 78/374444 (7.61) [7.1%] | 39/374712 (3.8) [3.5%] |
| NCT01370005 | 0 | <10/69992 (0.52) [0.1%] | 64/66580 (35.11) [7.8%] | 54/67092 (29.4) [6.5%] | 16/69476 (8.41) [1.9%] | 18/69120 (9.51) [2.2%] |
| NCT01381900 | 0 | 0 | 55/79955 (0.07) [8.11%] | 0 | 13/83810 (0.02) [1.92%] | 32/82595 (0.04) [4.72%] |
| NCT01422876 | 0 | 0 | 156/422226 (13.49) [11.1%] | 20/458474 (1.59) [1.4%] | 46/454983 (3.69) [3.3%] | 35/454911 (2.81) [2.5%] |
| NCT01438814 | 0 | 0 | 230/49168 (170.86) [33.4%] | <10/66700 (4.38) [1.2%] | 10/66749 (5.47) [1.5%] | <10/67018 (1.09) [0.3%] |
| NCT01558271 | 0 | 0 | 163/129809 (0.13) [33.13%] | 0 | 18/168170 (0.01) [3.66%] | <10/168987 (0) [0.81%] |
| NCT01606007 | 0 | 0 | 52/79559 (23.87) [9.9%] | 0 | <10/83523 (3.94) [1.7%] | <10/83873 (3.05) [1.3%] |
| NCT01619059 | 0 | 0 | 0 | 0 | <10/49692 (0.02) [2.95%] | 0 |
| NCT01621178 | 0 | 0 | 163/137947 (43.16) [28.2%] | 14/175865 (2.91) [2.4%] | 95/161613 (21.47) [16.5%] | <10/178224 (1.23) [1%] |
| NCT01624259 | 0 | 0 | 199/76228 (95.35) [33.2%] | <10/106240 (0.34) [0.2%] | 14/105161 (4.86) [2.3%] | <10/105810 (1.38) [0.7%] |
| NCT01632007 | 0 | 0 | 36/38793 (0.09) [14.69%] | 0 | <10/41762 (0.02) [2.86%] | 0 |
| NCT01632163 | 0 | 0 | 69/33345 (0.21) [27.49%] | <10/42582 (0.02) [2.79%] | <10/42555 (0.02) [2.79%] | <10/43153 (0) [0.4%] |
| NCT01648582 | 0 | 0 | 190/221750 (0.09) [24.55%] | 0 | 48/274535 (0.02) [6.2%] | 50/275863 (0.02) [6.46%] |
| NCT01708902 | 0 | 0 | 119/105485 (41.2) [16.2%] | <10/117240 (2.8) [1.2%] | 10/116899 (3.12) [1.4%] | <10/117469 (0.93) [0.4%] |
| NCT01719003 | 0 | 0 | 193/200549 (35.15) [14.1%] | 14/217825 (2.35) [1%] | 25/217927 (4.19) [1.8%] | 21/217531 (3.53) [1.5%] |
| NCT01734785 | 0 | 0 | 73/79111 (33.7) [21.9%] | 11/92413 (4.35) [3.3%] | 27/89407 (11.03) [8.1%] | <10/93385 (2.35) [1.8%] |
| NCT01768559 | 0 | 0 | 184/134203 (0.14) [20.63%] | 209/133959 (0.16) [23.43%] | 34/155422 (0.02) [3.81%] | 28/155029 (0.02) [3.14%] |
| NCT01769378 | 0 | 0 | 57/40926 (50.87) [19%] | <10/48169 (1.52) [0.7%] | <10/47864 (5.34) [2.3%] | 0 |
| NCT01778049 | 0 | 0 | 64/160059 (14.6) [13.3%] | <10/171960 (1.49) [1.5%] | 27/168925 (5.84) [5.6%] | 17/169835 (3.66) [3.5%] |
| NCT01792518 | 0 | 0 | 32/58078 (20.12) [8.9%] | 33/57230 (21.06) [9.2%] | 25/58632 (15.57) [6.9%] | <10/59994 (3.04) [1.4%] |
| NCT01798706 | 0 | 0 | 112/38422 (0.29) [34.46%] | 0 | 18/50840 (0.04) [5.54%] | <10/51112 (0.01) [1.85%] |
| NCT01809327 | 0 | <10/211272 (0) [0.08%] | 138/195426 (0.07) [11.64%] | 0 | 27/209682 (0.01) [2.28%] | 29/208541 (0.01) [2.45%] |
| NCT01890122 | 0 | 0 | 51/99445 (18.73) [7.9%] | 0 | 19/103659 (6.69) [2.9%] | <10/104290 (0.7) [0.3%] |
| NCT01989754 | 0 | <10/3919604 (0) [0.15%] | 245/3850467 (0.01) [4.21%] | <10/3920213 (0) [0.02%] | 1445/3437327 (0.04) [24.86%] | 162/3864836 (0) [2.79%] |
| NCT02025907 | 0 | 0 | 24/34196 (0.07) [11.01%] | 0 | <10/36648 (0.01) [1.38%] | <10/35856 (0.02) [3.67%] |
| NCT02065791 | 0 | 13/3585982 (0) [0.3%] | 1066/3032910 (0.04) [24.22%] | <10/3585007 (0) [0.09%] | 1487/3027828 (0.05) [33.79%] | 489/3357116 (0.01) [11.11%] |
| NCT02152371 | 0 | 0 | 67/46782 (52.31) [22.3%] | <10/55619 (0.66) [0.3%] | 16/54248 (10.77) [5.3%] | <10/55678 (1.31) [0.7%] |
| NCT02182830 | 0 | 0 | 20/22614 (0.09) [12.2%] | 0 | <10/24119 (0.03) [4.27%] | <10/23577 (0.04) [5.49%] |
| NCT02284893 | 0 | 0 | 46/127748 (13.15) [10.9%] | 0 | 15/134972 (4.06) [3.5%] | 0 |
| NCT02453555 | 0 | 0 | 57/84311 (0.07) [20.73%] | 0 | <10/95271 (0.01) [3.27%] | <10/95140 (0.01) [2.91%] |
| NCT02471404 | 0 | 0 | 163/300024 (19.84) [18%] | 0 | 92/320991 (10.47) [10.2%] | 28/333030 (3.07) [3.1%] |
| NCT02489968 | 0 | 0 | 72/151270 (17.38) [16.1%] | <10/173967 (0.63) [0.7%] | 17/170786 (3.64) [3.8%] | 10/171606 (2.13) [2.2%] |
| NCT02597049 | 0 | 0 | 105/57554 (66.64) [24.8%] | <10/71114 (0.51) [0.2%] | 14/70204 (7.28) [3.3%] | 10/70313 (5.19) [2.4%] |
| NCT02681094 | 0 | 0 | 66/142151 (16.96) [7.4%] | 0 | 23/146967 (5.72) [2.6%] | <10/147551 (2.23) [1%] |
| NCT02750410 | 0 | 0 | 61/38217 (0.16) [38.36%] | 0 | <10/53133 (0.01) [4.4%] | <10/53747 (0.01) [2.52%] |

######

###### Cells contain events/person-time in days [event rates per 100 person-years] and the percentage of participants enrolled experiencing the event

# eMethods

## Search strategy

Full search terms used in each database are shown in <https://bmjopen.bmj.com/content/bmjopen/12/10/e066491.full.pdf?with-ds=yes>

## Data extraction

WHO ATC drug names, drug doses and regimens were extracted from text strings obtained from clinicaltrials.gov and published documents (papers and clinical study reports). HbA1c results were extracted from clinicaltrials.gov where available or clinical documents if not. Outcomes were captured at arm, contrast and/or pre-and post intervention level. Age and sex at baseline were obtained from published documents, first by reading the tables into software for processing tabular data (https://www.tabletidier.org) then into R. All results were checked manually. For MACE, results were obtained via manual extraction from published documents. For IPD trials, data were cleaned and harmonised in the Vivli repository.

## Statistical analysis

All analyses requiring access to IPD were conducted within the Vivli safe haven/trusted research environment (hereafter, Vivli). All other analyses were conducted on machines within the University of Glasgow (hereafter, local). All analyses involving IPD were conducted in two stages. First trial-level analyses of IPD were produced within Vivli and summaries were exported. Secondly, these exported summaries were combined locally with aggregate level data. This two-step approach was chosen to meet the terms of the data sharing agreement, minimise the use of intensive computing resources within the Vivli environmentm, and finally to maximise resuse and ensure reproducibility.

## Baseline characteristics

In five of the IPD MACE trials, age was plotted within Vivli and found to be well-approximated with a normal distribution. Therfore, these were summarised using the mean and standard deviation. In the remaining MACE trial, age had been binned into three categories during the anonymisation process before it was provided to us, so the number of participants in each age category was summarised using simple counts. Sex was summarised as the number of participants in each trial and arm who were male and female.

As age in some of the IPD HbA1c trials did not follow a normal distribution, we summarised the distributions using empirical cumulative distribution functions (ECDFs). We estimated the ECDFs within each arm and sex and summarised these using the Ramer–Douglas–Peucker algorithm (https://cran.r-project.org/package=RDP), which reduces the number of points required to represent a curve or polygon. As a check on the summaries, they were plotted against the full ECDFs before being exported from Vivli. In stage two, we recreated full ECDFs by linear interpolation, and sampled from these to obtain age distributions.

For aggregate-level trials (both HbA1c and MACE) we estimated the age distribution from published summary statistics. For trials with age cut-offs below 20 years and above 100 years we assumed a normal distribution, otherwise we assumed a truncated normal distribution. We estimated the central tendency parameter ($\mu$) and dispersion parameter ($\sigma$) of the latter by numerical optimisation, based on the reported mean, standard deviation and upper and lower age limits. As, based on the IPD trials, age was only weakly associated with sex, we assumed the age distribution was the same for men and women. To obtain plots and summary statistics for the age distributions across trials we sampled from truncated normal/normal distributions setting the number of samples equal to the number of participants in each arm/sex stratum. As a check on this approach we repeated this method for the IPD trials comparing the results to the ECDF-derived distributions described above.

## Multi-level network meta-regression models (MLNR)

Each model was first fitted assuming identical (fixed) effects across trials for the same treatment. For all HbA1c analyses and selected MACE analyses, this assumption was then relaxed to allow exchangeable (random) effects. Main effects for the covariates and covariate-treatment interactions were assumed to be common across trials within the same drug class.

For IPD, data can be included either as the raw data or, equivalently, as outputs from trial-level models fitted to the data (ie the coefficients and variance-covariance matrices). We used the latter; this was possible due to a recent update to the multinma package. Within Vivli, for each of the IPD trials, we fitted trial-level regression models of each outcome on age, sex and treatment as well as age-treatment and sex-treatment interactions. For HbA1c we fitted linear regression models (additionally including HbA1c at baseline as a covariate). For the MACE outcomes we fitted Cox regression models. In both cases we exported the model outputs from the Vivli environment.

For HbA1c aggregate-level trials, the outcomes were modelled using arm-level data (the change in HbA1c in each arm and the accompanying standard error), contrast-level data (the difference between arms and standard error) or, rarely, as post-treatment estimates. For MACE aggregate-level trials the outcome was modelled as contrast level estimates (log-hazard ratios and standard errors for log-hazard ratios). For HbA1c, since the outcome is continuous, only the means for the covariates are required (ie the mean age, mean baseline HbA1c and the proportion of men in each trial/arm). As MACE is not modelled on a linear scale the full joint distribution of the covariates (age and sex) was used - the age distributions were represented using truncated normal distributions (obtained as described above) with sex represented as a Bernoulli distribution (with the parameter equal to the percentage of men in each trial arm). The correlation between these variables was assumed to be the same as that observed in trials for which we had IPD.

For MACE trials we also modelled, separately, subgroup data for age and sex alongside the IPD and aggregate level data. For the sex subgroup data, we assumed the same age distribution across sex strata. For the age subgroup data, we assumed the same proportion were male across age strata. As before, age was represented using truncated normal distributions, with the same $\mu$ and $\sigma$ as the earlier models without subgroup data, but with cut-points equal to the stratum-defining age limits (eg $= 60 $\sigma=10$, lower = 20 and upper = 69 for a trial with an age subgroup cut-point of 70). Finally, in order to estimate age-sex specific efficacy for MACE at drug-class level, we labelled all treatment arms according to the drug class (ignoring specific drug/dose) and re-ran the model with IPD, aggregate level and sex-subgroup data.

For all models we ran four chains for 2,000 iterations each, and checked for divergent transitions, model convergence (visually using caterpillar plots and using the Gelman-Ruben statistic) and autocorrelation using the R shinystan package. These models were run on a high-performance computing environment within the University of Glasgow. See appendix for additional details of modelling and github for the data and multinma model code. The first 1,000 iterations from each chain were discarded, leaving 4,000 samples. Models were summarised using the mean and 95% credible interval, obtained as the 2.5th and 97.5th quantiles of the 4,000 samples.

## Sensitivity analyses of ML-NMR findings

For the HbA1c analyses we conducted a range of sensitivity analyses including:-

- Limiting the meta-analysis to IPD trials (which makes no assumptions about the age-sex distributions in the aggregate level trials)
- Limiting the trials to those with >= 26 weeks follow-up
- Replacing coefficients from trial-level models using last observation carried forward (LOCF) with models using baseline observation carried forward (BOCF)

We estimated the age-treatment and sex-treatment interactions for MACE for a range of sensitivity analyses. We ran models dropping/downgrading (from IPD to subgroup data where this was available) each of the 23 trials in turn. However, we only present results for each of the following three trials:- i) the single IPD trial for DPP-4s, ii) the single IPD trial for GLP-1s and iii) one of the four IPD trials for SGLT-2. We do so in order to simplify the presentation, as on dropping/downgrading each of the other trials there were no differences in the results when compared to the “all trials included” analyses (see model outputs in the project github repository).

All of the age-treatment and sex-treatment results which analysed subgrup data were obtained using fixed effects models as it is not currently possible to fit random effects models with subgroup data in the multinma package. Nonetheless, similar results were found in fixed and random effects models for the models which did not include subgroup data (see main manuscript).

## Examinination of non-linearity in the age-treatment interactions HbA1c

Non-linearity in the age-treatment interactions was examined by visual inspection of the residuals of the model for each trial, and by fitting a LOWESS smoother to the residuals. As residual plots contain individual-level data, exporting these from the Vivli environment may have been challenging. Therefore, for each trial, we also fitted models of HbA1c on baseline HbA1c, sex, age and the age-treatment and sex-treatment interactions. For age (and the age-treatment interaction) we used restricted cubic splines with knots at 40, 60, 60 and 75 to allow for non-linearity. We exported the model outputs from the Vivli environment. For each trial, we obtained curves for the age-treatment interaction (not including the main treatment effect), sampling from a multivariate normal distribution (taking the coefficient values and variance-covarance matrix elements corresponding to the age-treatment interaction parameters) and applying these to transformed age values for ages 40 through 75 (see github Rcode script). We then fitted a simple linear model through these points to compare the curves to a linear approximation.

## Examination of non-linearity in the age-treatment interactions for MACE

As for the HbA1c trials, for the MACE trials we also examined for non-linearity in the age-treatment interactions by visual inspection of the (Martingale) residuals of the model for each trial, and by fitting a lowess smoother to the residuals). For two of the canagliflozin trials there was some suggestion of non-linearity. However, the apparent patterns of non-linearity were not consistent; There was no consistency across two drug arms (with different doses) in the same trial, nor for the same drug across the two different trials. As for the HbA1c analysis, we also fitted models with restricted cubic splines. We fitted Cox models of MACE on sex, age and the age-treatment and sex-treatment interactions using restricted cubic spline for age. For the MACE trials, we fixed the knots at the 5th, 27th, 50th, 72nd and 95th centiles.

# eResults

## Non-linearity in the age-treatment interactions for HbA1c

Consistent with the residual plots, there was no evidence of non-linearity in the age-treatment interaction for HBA1c. For MACE, there was some evidence of non-linearity for one of the canagliflozin trials; the tendency towards increased efficacy with age (trial NCT01989754 below) appeared to be greater at older ages. Nonetheless, the overall pattern was reasonably approximated with a straight line within the range of the data. We would urge caution in treating the relationship as non-linear as compared to our main analysis this is anti-conservative, as it suggests yet greater efficacy at older ages.

## Models without the use of IPD

Using the ML-NMR it is not only the IPD trials which contribute to the estimation of the age-treatment and sex-treatment interactions, both IPD and aggregate level trials do; the same individual-level model is fitted to both types of trial, avoiding aggregation bias, all estimates are within trial estimate of heterogeneity. This is possible because it the model integrates over the distribution of the covariates for the aggregate level trials.

Therefore, it is theoretically possible to fit the models without any individual-level data. However, since considerably more information can be obtained from individual-level data than in the aggregate data, a large number of aggregate level trials (which are sufficiently heterogeneous in terms of age and sex) would be needed to achieve reasonably precise estimates.

For illustrative purposes we re-fitted the models replacing the trial IPD with aggregate level data (the age and sex distributions and the HBA1c-results for each arm). The models did not converge, and so could not be used for inferences. For MACE, where there was only 23 trials we also re-ran the analysis substituting aggregate-level (contrast) data for IPD. In this case the models did converge (although with some divergent transitions in the case of the random effects model). However, without IPD the estimates were so wide as to be completely unusable.

## Missingness

Of 103 IPD HbA1c trials, none had missing sex data. Twelve had missing age data; of these nine had missing age data for <1% participants, with the remaining three having missing age data for 1.29%, 1.34% and 1.95% respectively. Nine had no HbA1c at follow-up; of these six had missing HbA1c data for less than 1% of participants, with the remaining having missing HbA1c data for 1.11%, 1.17% and 4.81% respectively. The trials/participants with missing HbA1c at follow-up were a subset of the trials/participants with missing age data. Of the six IPD MACE trials three had missing age data; there were three (0.06%), two (0.02%) and one (0.01%) of participants with missing age data respectively. Missing age data was due to the trial sponsors redaction algorithm. We did not obtain data on missingness in for the aggregate-level trials.
